# Theoretical study on the conformation-dependent charge transfer of the excited state of dopamine

**DOI:** 10.1016/j.heliyon.2025.e42058

**Published:** 2025-01-17

**Authors:** Huan An, Asiya, Gulmire Yaermaimaiti, Bumaliya Abulimiti, Mei Xiang, Xiaoning Wang

**Affiliations:** a Xinjiang Key Laboratory for Luminescence Minerals and Optical Functional Materials, School of Physics and Electronic Engineering, Xinjiang Normal University, Urumqi, 830054, China; b School of Chemistry and Chemical Engineering, Xinjiang Normal University, Urumqi, 830054, China

**Keywords:** Dopamine, Conformation, Excited-state, Charge transfer, Theoretical research

## Abstract

Clarifying the relationship between conformation and charge transfer is crucial for understanding the functional mechanisms of molecules in organisms. Theoretical calculations of dopamine, N,N-dimethyldopamine, and N,N-dihydroxydopamine were related to conformation and charge transfer, and the charge transfer of the excited state was clearly characterized. First, the stable configuration of the ground state of the molecule was optimized, and the potential energy of the ionic state was scanned to select all conformations, except for the chiral problem. Subsequently, the CAM-B3LYP/aug-cc-pVTZ method was used to excite the molecule, and the excitation types of the first five excited states of the molecule were discussed. Finally, the charge transfer of the molecule was calculated, and the charge transfer of the different conformations was analyzed in detail. Through charge transfer, it has been speculated that dopamine molecules exist in a coiled state in an organism and interact with water to form N,N-dihydroxydopamine, or hydrogen bonds for better information transmission.

## Graphical abstract

The N,N-dihydroxydopamine has better CT ability than dopamine. It is guessed that in organism, dopamine forms N,N-dihydroxydopamine, or dopamine forms hydrogen bonds with water to achieve better signal transmission.

**Figure:**
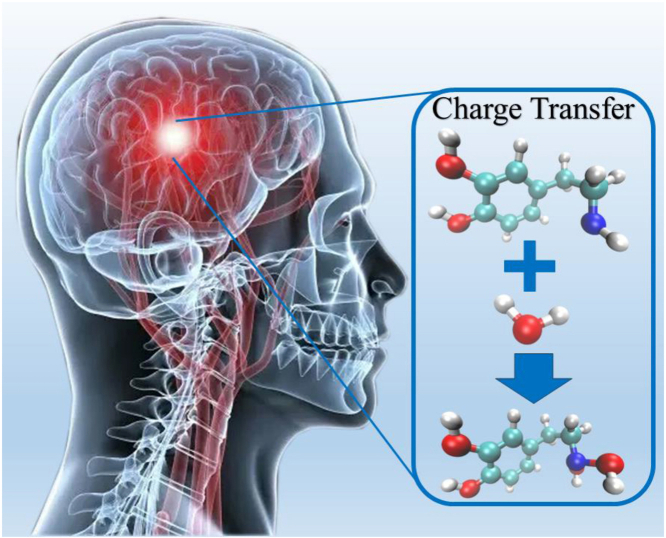

## 1 Introduction

Charge transfer (CT) is a crucial reaction ubiquitous in biological systems, including DNA and proteins, and plays a role in light collection and functional devices [1]. Excited-state intramolecular charge transfer (ICT) process refers to the CT process from an electron donor group to an acceptor group within a molecule upon excitation [2]. Molecular ICT is a prominent topic in photophysics and photochemistry [[3], [4], [5], [6], [7]]. With scientific advancements, the significance of molecular isomer recognition and its dynamics in CT research have become evident. Different molecular conformations exhibit different CT capabilities; thus, an in-depth understanding of the conformation and CT process is essential in the fields of chemistry and biology. For example, the functions of biological molecules such as DNA, enzymes, and proteins have been studied [[8], [9], [10], [11], [12], [13], [14], [15]].

To observe the excited-state CT process in real time, scholars have developed various time-resolved mass spectrometry techniques based on femtosecond pump-probe methods, time-resolved photoelectron imaging techniques, time-resolved fluorescence upconversion techniques, and time-resolved transient absorption spectroscopy techniques. For instance, Hofkens et al. [16] utilized time-resolved fluorescence spectroscopy to investigate the impact of solvent polarity, substitution sites, and solution viscosity on the ICT of phenylethylene molecular derivatives. Ju et al. [17] designed and tested two D-B-A compounds (TRD and TTRD) by UV–visible spectroscopy, steady-state emission spectroscopy, X-ray diffraction, electrochemical measurements, photovoltaic performance, and theoretical calculations. They found that these compounds exhibited large Stokes shifts, indicating the formation of ICT states in the excited state. Weber et al. [18] employed femtosecond time-resolved spectroscopy to explore the conformation-dependent charge delocalization process during the CT of 1,3,5-trimethylhexylhydroxy-1,3,5-triazine molecules. Shi et al. [19] used femtosecond transient absorption spectroscopy and density functional theory (DFT) to investigate the effect of the solvent hydrogen supply capacity on ultrafast ICT. Kwak et al. [20] utilized DFT and time-dependent DFT (TD-DFT) methods combined with molecular dynamics and quantum mechanics/molecular mechanics simulations to study the charge transfer of three Au (III) complexes in thin films. The HOMO and LUMO were effectively separated, and the first singlet excited state (S_1_) and first triplet excited state (T_1_) exhibited ligand-to-ligand charge-transfer characteristics. Li et al. [21] calculated the vertical excitation energy using the TD-DFT method and combined nuclear magnetic resonance, ultraviolet–visible–infrared spectroscopy, and fluorescence spectroscopy to characterize the hydrogen bonds between molecules that affect the CT process. Several factors influence the CT process, such as solvent polarity, substitution site, solvent viscosity, molecular structure/conformation, solvent hydrogen supply capacity, substitution effect, and intermolecular hydrogen bonds. Currently, great progress has been made in the study of the ICT process of molecular excited states; however, the relationship between conformation and CT cannot be well explained for the time being.

Dopamine is one of the main neurotransmitters that significantly influence information transmission in organisms. In recent years, dopamine research has primarily focused on detection methods. One widely used approach is neurotransmitter fluorescence detection, which enables the monitoring of synaptic transmission with a response time of only a few seconds and single-cell precision [22]. After neurotransmitter binding, fluorescence emission is recorded by optical transducers, such as fiber photometry [22], bio-photometry [23], or characterization bio-devices [24]. Specific in vivo biosensors can be inserted into animal brains using implantable devices, viral vectors, or specific cell expression [25]. Natural receptors of dopamine, which belong to the G protein-coupled receptor (GPCRs) family, offer optimal selectivity and specificity [26,27]. Recently, natural receptors were used to recognize elements within Field Effect Transistors (FET) using receptor-containing nanovesicles with functional GPCR, arousing interest in in vitro biosensors, as well [22,28,29].

However, the study of dopamine conformation and charge transfer is important. Studying the relationship between conformation and CT can help understand the biological mechanisms of dopamine. In addition, when a molecule is excited to the Rydberg state, the electrons are positioned far away from the molecular ion nucleus, resulting in a structure that closely resembles the cationic state [30]. As a result, the excitation energy of the Rydberg state is not significantly affected by the molecule's vibrational state but is notably influenced by its molecular geometry. In this study, the ground state (S_0_) stable configurations of dopamine and its derivatives (N,N-dimethyldopamine and N,N-dihydroxydopamine) were calculated using MP2 at the 6-311+G(d,p) basis set level. Subsequently, based on the structure of the S_0_, the potential energy of the molecular ion state was scanned to determine the global and local minimum conformations. The MP2/6-311+G(d,p) basis set was used to optimize the structure of the molecular ion state (D_0_), and the potential energy of D_0_ was analyzed. Finally, the CAM-B3LYP/aug-cc-PVTZ method was utilized to excite different conformations of the molecule, the properties of the five excited states were analyzed, and the CT situation was described. The findings of this study provide valuable insights into the electronic excited-state structure and kinetic mechanisms of dopamine and its derivatives. Moreover, they provide guidance for investigating the conformational structures and properties of more complex amino acid and peptide systems. In addition, our results lay the foundation for a comprehensive analysis of the signal transmission mechanisms of neurotransmitters.

## 2 Materials and method

The theoretical calculations in this work were completed using Gaussian 16 [31] quantum computing software. Initially, the MP2/6-311+G(d,p) [32,33] method was used to optimize the S_0_ structure of dopamine, N,N-dimethyldopamine, and N,N-dihydroxydopamine molecules to obtain a stable configuration. Based on this structure, Molclus 1.9.9.7 [34] and Gaussian 16 were used to scan the conformations of the three molecular D_0_. Special attention was paid to the strong flexibility of the molecular system; thus, the scanning conformation was divided into two steps. First, the C_3_-C_14_ bond of the molecule was rotated by 5°, and 72 molecular configurations were obtained. Molecular configurations with the smallest global and local energies were selected. Second, the C_14_-C_17_ and C_17_-N_20_ bonds of the molecule were rotated simultaneously with a step size of 10°. Finally, the molecular configuration with the smallest global and local energy was selected. The ionic conformation identified again at the MP2/6-311+G(d,p) basis set level was optimized, and the molecular configuration was assigned and discussed. Finally, the CAM-B3LYP/aug-cc-PVTZ [35] method was used to excite the molecule, and the Multiwfn 3.7 [36,37] software package developed by Lu Tian's team was used to analyze the characteristics of the five excited states in detail; the excitation types were assigned, and the CT of different conformations was analyzed. It should be noted that no imaginary frequencies were observed in the calculated frequencies of all structures, indicating the validity of the calculations.

## 3 Results and discussion

### 3.1 The ground state and ionic structure of the molecule

**Fig. 1:**
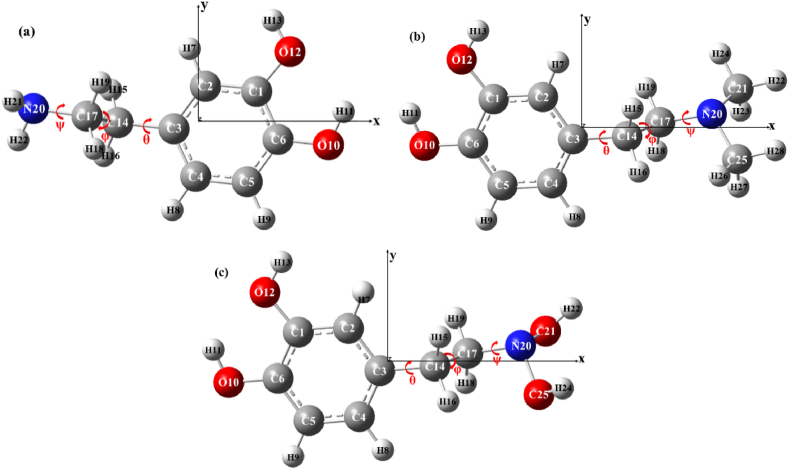
The ground state structures of (a) dopamine, (b) N,N-dimethyldopamine, and (c) N,N-dihydroxydopamine. The black arrow is Cartesian coordinate system, and the Z axis is vertical outward. The red arrow indicates the direction of key rotation. The calculation method and basis set are MP2/6-311+G(d,p).

To analyze the conformation-dependent CT process, the MP2 and 6-311+G(d,p) basis sets were used to optimize the ground-state stable configurations of dopamine, N,N-dimethylethylamine, and N,N-dihydroxydopamine, as illustrated in Fig. 1. The corresponding ground state energies are −14018.00984 eV, −16149.78402 eV, and −18099.71975 eV, respectively. The black arrow is the Cartesian coordinate system, with the z-axis perpendicular to the exterior, and the red arrow is the key rotation position for the subsequent scanning of the ion potential energy, represented by θ, φ, and ψ, respectively. It is essential to note that the molecular conformation is named based on the rotation angles of three bonds, denoted as (θ,φ,ψ).

**Fig. 2:**
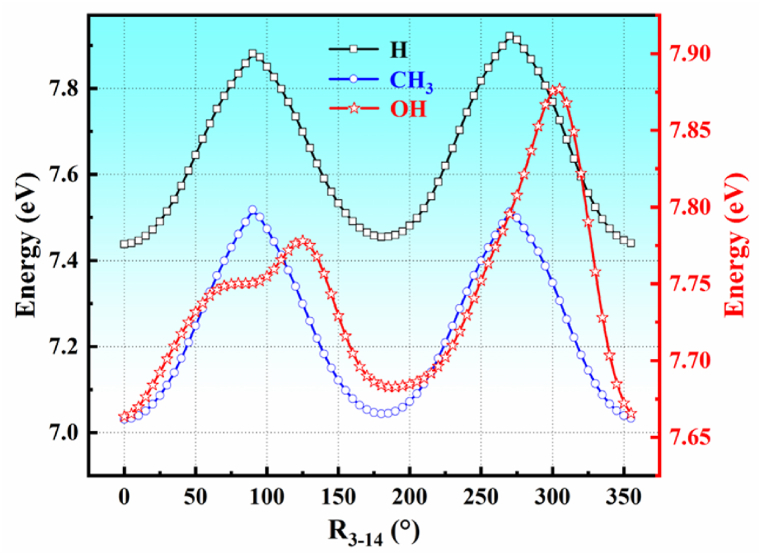
The potential energy of (a) dopamine, (b) N,N-dimethyldopamine, and (c) N,N-dihydroxydopamine obtained rotating around the C_3_-C_14_ bond. The black and blue lines refer to the left coordinates, and the red lines refer to the right coordinates. The calculation method and basis set are B3LYP/6-311+G(d,p).

**Fig. 3:**
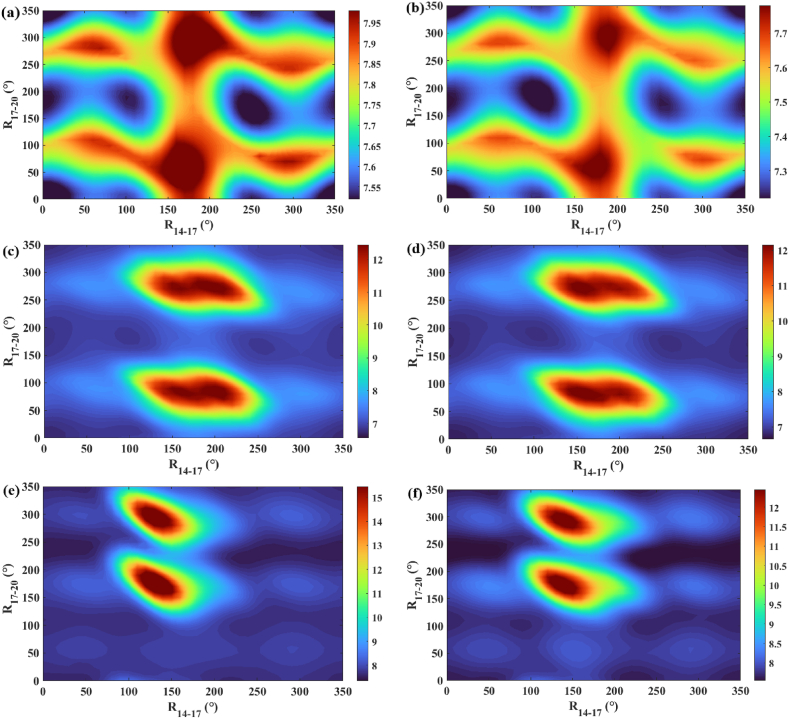
The potential energy of the ionic states of (a–b) dopamine, (c–d) N,N-dimethyldopamine, and (e–f) N,N-dihydroxydopamine rotating simultaneously around the C_14_-C_17_ and C_17_-N_20_ bonds. The calculation method and basis set are B3LYP/6-31G.

**Fig. 4:**
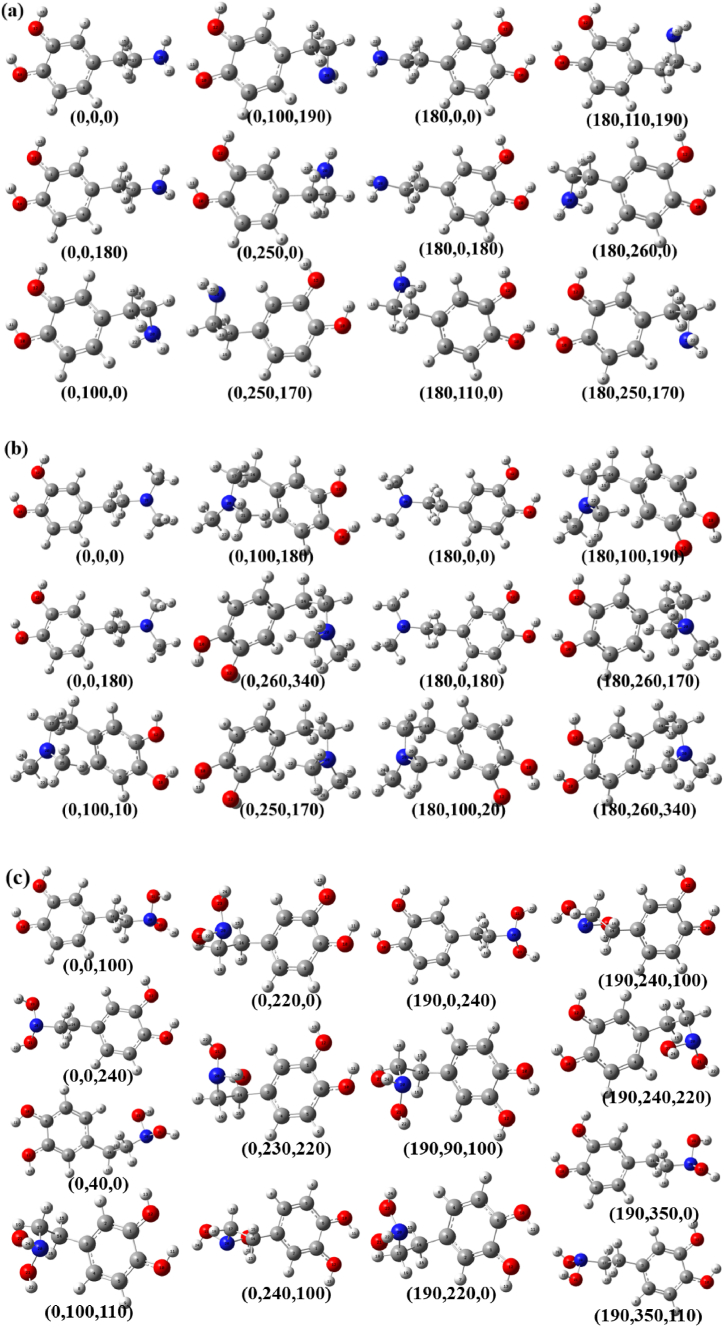
Conformations of (a) dopamine, (b) N,N-dimethyldopamine, (c) N,N-dihydroxydopamine. As described above, the (θ,φ,ψ) method is named. The calculation method and basis set are MP2/6-311+G(d,p).

Based on the above-ground state stable conformation, the potential energy of D_0_ for the three molecules was scanned. Dopamine, N,N-dimethylethylamine, and N,N-dihydroxydopamine exhibit significant flexibility (molecular structure and flexibility); therefore, this process is completed in two steps. First, the C_3_-C_14_ bond of the molecule was rotated in 5 °steps, resulting in 72 molecular configurations. From these, the two configurations with the lowest energies were selected, as illustrated in Fig. 2. Second, we continued to rotate the C_14_-N_17_ and C_17_-N_20_ bonds of the molecule at the same time with a step size of 10°, and finally selected the molecular configuration with the smallest global and local energies, as shown in Fig. 3. The first-step calculation utilized the B3LYP/6-311+G(d,p) basis set, whereas the second step was performed using the B3LYP/6-31G basis set. Through the above two-step process, 12, 12, dopamine, N, N-dimethyldopamine, and N, N-dihydroxydopamine were obtained, respectively. To determine the accuracy of the conformations of the molecules, they were optimized using the MP2/6-311+G(d,p) basis set, as shown in Fig. 4. The total energy of the molecular ion state is extracted as shown in Table 1, and the energy here is based on the ground state energy. By analyzing the energy, it can be found that many energies are close or equal. For example, the energies of dopamine conformations with θ angles of 0° and 180° are almost equal or close. Further analysis of their conformations indicated that the same energy was due to their chiral symmetry. Because chiral molecules occupy almost the same electronic orbitals and exhibit almost the same CT process, we temporarily attributed chiral molecules to a conformation. To date, dopamine has exhibited only six conformations: (0,0,0), (0,0,180), (0,100,0), (0,100,190), (0,250,0) and (0,250,170). Similar to N,N-dimethyldopamine, there are four conformations: (0,0,0), (0,0,180), (0,100,10), and (0,260,340). N, N-Dihydroxydopamine presents seven conformations: (0,0,100), (0,0,240), (0,40,0), (0,100,110), (0,220,0), (0,240,100), and (190,240,220).

**Table 1:** The energy corresponding to the ionic conformations of dopamine, N,N-dimethyldopamine, and N,N-dihydroxydopamine. The calculation method and basis set are: MP2/6-311+G(d,p).

Dopamine		N,N-Dimethyldopamine		N,N-Dihydroxydopamine	
Conformation	Energy (eV)	Conformation	Energy (eV)	Conformation	Energy (eV)
(0,0,0)	8.0465	(0,0,0)	8.1899	(0,0,1000)	8.0545
(0,0,180)	8.0288	(0,0,180)	7.6513	(0,0,240)	7.9789
(0,100,0)	8.0789	(0,100,10)	7.4312	(0,40,0)	8.0449
(0,100,190)	7.8841	(0,100,180)	7.4312	(0,100,110)	7.9859
(0,250,0)	8.0534	(0,260,340)	7.4505	(0,220,0)	7.9662
(0,250,170)	7.8579	(0,250,170)	7.4505	(0,230,220)	7.9390
				(0,240,100)	7.7903

(180,0,0)	8.0465	(180,0,0)	8.1902	(190,0,240)	7.9789
(180,0,180)	8.0251	(180,0,180)	7.6512	(190,90,100)	7.9662
(180,110,0)	8.0534	(180,100,20)	7.4505	(190,220,0)	7.9859
(180,110,190)	7.8579	(180,100,190)	7.4505	(190,240,100)	7.9297
(180,250,170)	8.0789	(180,260,170)	7.4312	(190,240,220)	7.8167
(180,260,0)	7.8841	(180,260,340)	7.4312	(190,350,0)	8.0545
				(190,350,110)	8.0526

In addition, the Boltzmann distributions of the ion states at different vibrational temperatures were calculated, as shown in Supplementary Material S2. The (190,240,220) conformation of N,N-dihydroxydopamine had a larger proportion, and N,N-dihydroxydopamine may exist in the form of crimp folding.

### 3.2 Excited-state properties of molecules

Studying the excited-state properties of molecules is important for understanding their properties and structures. The focus of this study is to investigate the conformation-dependent CT of molecules. Because CT primarily occurs in the excited state, it is essential to understand the characteristics of molecules in this state. Fortunately, Lu's group [36] summarized the overlap function (*Sr*) of hole and electron, the distance between the center of mass of hole and electron (*D*), the overall average distribution width of electron and hole (*H*), the overall distribution width of hole or electron (*σ*_*h*_ or *σ*_*e*_), the separation degree of hole and electron (*t*), the hole delocalization index (*HDI*), the electron delocalization index (*EDI*) and adiabatic excitation energies (these parameters are called excited-state parameters in the following section). The excited state can be detected more clearly by considering the excited-state parameters. A smaller *Sr* value indicates a more significant separation between the holes and electrons, whereas a larger *D* index suggests a more pronounced separation. In addition, smaller *HDI* (*EDI*) values indicate a more uniform distribution of holes (electrons). Therefore, it is simple to analyze the electronically excited state using the excited-state index and the corresponding hole-electron diagram, Chole–Cele, and *Sr* function diagrams.

**Fig. 5:**
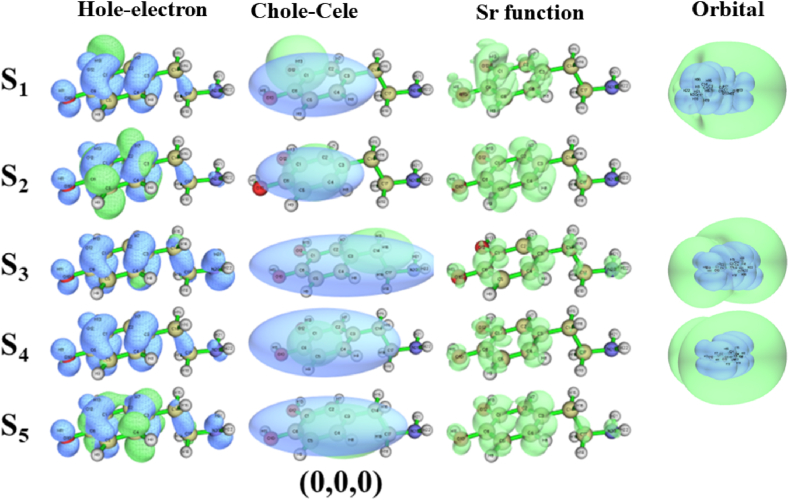
The hole-electron diagram, Chole-Cele diagram, *Sr* function diagram, and molecular orbital of the 5 excited states of dopamine (0,0,0) conformation. The calculation method and basis set are CAM-B3LYP/aug-cc-pVTZ.

First, the properties of the five excited states of dopamine are discussed. Considering the possibility of Rydberg states occurring during excitation, the excited states of the six dopamine conformations were calculated using the CAM-B3LYP/aug-cc-pVTZ basis set. To ensure the accuracy of the calculation, we calculated the first ten excited states. The excited-state parameters of the first five excited states were calculated using Multiwfn 3.7, as detailed in Table S5 (Supplementary Material), and the hole-electron, Chole-Cele, and *Sr* function diagrams are shown in Fig. 5. The excited-state parameters for the (0,0,0) conformation are listed in Table S5. Examining the S_0_→S_1_ and S_0_→S_3_ excitations, the large *D* index suggests a significant separation between holes and electrons, possibly indicating CT excitation. Combined with the Chole-Cele diagram in Fig. 5, the center positions of holes and electrons are obviously shifted, which also indicates that CT excitation has occurred in the S_1_ and S_3_ states. The hole-electron diagram of the S_1_ state is observed (Fig. 5), most of the holes are located in the phenyl, and the spherical electron cloud is distributed in O_12_, indicating that the S_0_→S_1_ belongs to the CT excitation of π→n∗. The hole-electron diagram of the S_3_ state shows that phenyl occupies a large number of holes, and the electron cloud with σ-characteristics is distributed on C_14_, therefore, the S_0_→S_3_ belongs to the CT excitation of π→σ∗. Subsequently, we explored the S_0_→S_2_ and S_0_→S_5_ excitations. They have a large *Sr* index and a small *D* index, and the *t* index is negative, indicating that holes and electrons are more concentrated. The Chole-Cele diagram in Fig. 5 also shows that the centers of the holes and electrons are basically coincident, which is judged to be local excitation. The hole-electron diagrams of the S_2_ and S_5_ states (Fig. 5) show that a number of π-characterized holes are distributed on the phenyl, and the electrons of the S_2_ state are also distributed on the phenyl (showing a δ-characterize), the phenyl of the S_5_ state occupies the π-characterized electrons. Therefore, S_0_→S_2_ represents the local excitation of π→δ∗, and S_0_→S_5_ reflects the local excitation of π→π∗. Finally, the S_0_→S_4_ excitation displays a small D index and a notably negative t index, with holes and electrons mainly distributed on the phenyl, as indicated by the Chole-Cele diagram in Fig. 5. The hole-electron diagram (Fig. 5) illustrates that both holes and electrons are distributed on the phenyl, the S_0_→S_4_ belongs to the local excitation of π→π∗. In addition, the *HDI* values are much larger than the *EHI* values of S_0_→S_1_, S_0_→S_3,_ and S_0_→S_4_, indicating that the electrons are sufficiently dispersed. From the molecular orbital of Fig. 5, it can be seen that they belong to the Rydberg states. Molecular orbital (Fig. 5) quantum defect *δ* (*δ* for second-group elements is 0.9–1.2 for s orbital, 0.3–0.5 for p orbital, and approximately 0 for d orbital [[37], [38]].) indicates that the S_1_, S_3,_ and S_4_ states correspond to the 3s, 3p_x_ and 3p_y_ states, respectively. In a previous study [39], we introduced a detailed analytical method for the Rydberg state.

**Fig. 6:**
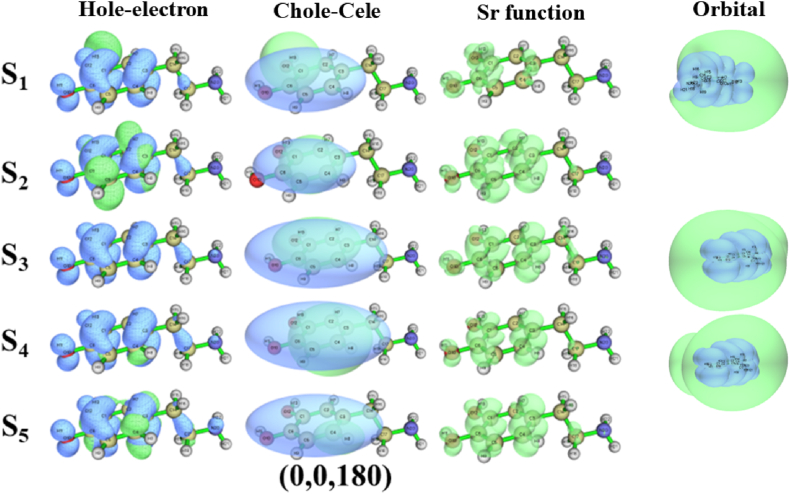
The hole-electron diagram, Chole-Cele diagram, *Sr* function diagram, and molecular orbital of the five excited states of the dopamine (0,0,180) conformation. The calculation method and basis set are CAM-B3LYP/aug-cc-pVTZ.

Judging the (0,0,180) conformation, the excitation process of S_0_→S_1_ has the characteristics of a small *Sr* index, a large *D* index, and *t* > 0, indicating CT excitation combined with the Chole-Cele diagram (Fig. 6). In the hole-electron diagram of the S_1_ state in Fig. 6, the hole exhibits prominent π-characteristic, and the electron is spherically distributed in O_12_, the S_0_→S_1_ is a CT excitation of π→n∗. The *Sr* index, *D* index, *t* index, and Chole-Cele diagram (Fig. 6) indicate that S_0_→S_2_, S_0_→S_3_, S_0_→S_4_, and S_0_→S_5_ are local excitations. At the same time, the *HDI* index, *EDI* index, *δ* index, and molecular orbital indicate that the S_1_, S_3_, and S_4_ states correspond to the 3s, 3p_x_, and 3p_y_ states.

Using this method, we identified the first five excited state types of dopamine, N,N-dimethyldopamine, and N,N-dihydroxydopamine, as shown in Table 2, Table 3, Table 4. The detailed excited-state parameters and the corresponding hole-electron diagram, Chole-Cele diagram, *Sr* function diagram, and molecular orbitals are displayed in the Supplementary Material.

**Table 2:** The excited-state types of six conformations of dopamine. CT is charge transfer excitation and LE is local excitation. The calculation method and basis set are CAM-B3LYP/aug-cc-pVTZ.

Conformation		States	Transition	Conformation		States	Transition
(0,0,0)	S_0_→S_1_	3s	CT(π→n∗)	(0,0,180)	S_0_→S_1_	3s	CT(π→n∗)
	S_0_→S_2_		LE(π→δ∗)		S_0_→S_2_		LE(π→δ∗)
	S_0_→S_3_	3p_x_	CT(π→σ∗)		S_0_→S_3_	3p_x_	LE (π→π∗)
	S_0_→S_4_	3p_y_	LE (π→π∗)		S_0_→S_4_	3p_y_	LE (π→π∗)
	S_0_→S_5_		LE (π→π∗)		S_0_→S_5_		LE (π→π∗)
(0,100,0)	S_0_→S_1_	3s	CT(π→n∗)	(0,100,190)	S_0_→S_1_	3s	CT(π→n∗)
	S_0_→S_2_		LE(π→δ∗)		S_0_→S_2_		LE(π→δ∗)
	S_0_→S_3_	3p_x_	LE (π→π∗)		S_0_→S_3_	3p_x_	CT(π→n∗)
	S_0_→S_4_	3p_y_	LE (π→π∗)		S_0_→S_4_	3p_y_	LE (π→π∗)
	S_0_→S_5_		LE (π→π∗)		S_0_→S_5_	3p_z_	LE (π→π∗)
(0,250,0)	S_0_→S_1_	3s	CT(π→n∗)	(0,250,170)	S_0_→S_1_	3s	CT(π→n∗)
	S_0_→S_2_		LE(π→δ∗)		S_0_→S_2_		LE(π→δ∗)
	S_0_→S_3_	3p_x_	LE (π→π∗)		S_0_→S_3_	3p_x_	CT(π→σ∗)
	S_0_→S_4_	3p_y_	LE (π→σ∗)		S_0_→S_4_	3p_y_	LE (π→π∗)
	S_0_→S_5_		LE (π→π∗)		S_0_→S_5_		LE (π→π∗)

**Table 3:** Excited-state types of four conformations of N,N-dimethyldopamine. CT is charge transfer excitation and LE is local excitation. The calculation method and basis set are CAM-B3LYP/aug-cc-pVTZ.

Conformation		States	Transition	Conformation		States	Transition
(0,0,0)	S_0_→S_1_	3s	LE(σ→σ∗)	(0,0,180)	S_0_→S_1_	3s	CT(π→n∗)
	S_0_→S_2_	3p_y_	LE(σ→σ∗)		S_0_→S_2_	3p_x_	LE(n→δ∗) LE (π→δ∗)
	S_0_→S_3_	3p_z_	LE(σ→σ∗)		S_0_→S_3_	3p_y_	LE(n→δ∗) LE(π→δ∗)
	S_0_→S_4_		LE(σ→σ∗) LE (π→π∗)		S_0_→S_4_		LE(σ→σ∗) LE(π→π∗)
	S_0_→S_5_	3p_x_	LE(σ→σ∗) LE (π→δ∗)		S_0_→S_5_	3p_z_	LE(σ→σ∗) LE(π→π∗)
(0,100,10)	S_0_→S_1_	3s	LE(σ→σ∗)	(0,260,340)	S_0_→S_1_	3s	LE(σ→σ∗)
	S_0_→S_2_	3p_x_	CT(σ→δ∗)		S_0_→S_2_		CT(σ→δ∗) LE(π→δ∗)
	S_0_→S_3_		LE(σ→π∗) LE(π→π∗)		S_0_→S_3_	3p_y_	CT(π→π∗)
	S_0_→S_4_	3p_y_	LE (σ→π∗)		S_0_→S_4_	3p_z_	LE(σ→π∗) LE(π→π∗)
	S_0_→S_5_	3p_z_	LE(σ→π∗) LE(π→π∗)		S_0_→S_5_	3p_x_	CT(σ→δ∗) LE(π→δ∗)

**Table 4:** Excited-state types of seven conformations of N, N-dihydroxydopamine. CT is charge transfer excitation and LE is local excitation. The calculation method and basis set are CAM-B3LYP/aug-cc-pVTZ.

Conformation		States	Transition	Conformation		States	Transition
(0,0,100)	S_0_→S_1_	3s	CT(π→n∗)	(0,0,240)	S_0_→S_1_	3s	CT(π→n∗)
	S_0_→S_2_		LE(π→δ∗)		S_0_→S_2_		LE(π→δ∗)
	S_0_→S_3_	3p_z_	LE(π→π∗)		S_0_→S_3_	3p_y_	LE(π→π∗)
	S_0_→S_4_	3p_y_	LE(π→π∗)		S_0_→S_4_	3p_x_	LE(π→π∗)
	S_0_→S_5_	3p_x_	LE(π→π∗)		S_0_→S_5_	3p_z_	LE(π→π∗)
(0,40,0)	S_0_→S_1_	3s	CT(π→n∗)	(0,100,110)	S_0_→S_1_	3s	CT(π→n∗)
	S_0_→S_2_		LE(π→δ∗)		S_0_→S_2_		LE(π→δ∗)
	S_0_→S_3_	3p_z_	CT(π→π∗) CT(n→π∗)		S_0_→S_3_	3p_y_	CT(π→σ∗) CT(σ→σ∗)
	S_0_→S_4_	3p_y_	LE(π→π∗) LE(n→π∗)		S_0_→S_4_	3p_x_	LE(π→π∗)
	S_0_→S_5_	3p_x_	LE(π→π∗) LE(n→π∗)		S_0_→S_5_	3p_z_	LE(π→π∗)
(0,220,0)	S_0_→S_1_	3s	CT(π→n∗)	(0,240,100)	S_0_→S_1_	3s	CT(π→n∗)
	S_0_→S_2_		LE(π→δ∗)		S_0_→S_2_		LE(π→δ∗)
	S_0_→S_3_	3p_y_	LE(π→δ∗)		S_0_→S_3_	3p_x_	CT(π→σ∗)
	S_0_→S_4_	3p_x_	LE(π→σ∗)		S_0_→S_4_	3p_y_	LE(π→σ∗)
	S_0_→S_5_	3p_z_	LE(π→σ∗)		S_0_→S_5_	3p_z_	CT(π→n∗)
(190,240,220)	S_0_→S_1_	3s	CT(π→n∗)				
	S_0_→S_2_		LE(π→δ∗)				
	S_0_→S_3_	3p_x_	CT(π→n∗) CT(σ→n∗)				
	S_0_→S_4_	3p_y_	LE(π→σ∗)				
	S_0_→S_5_	3p_z_	LE(π→π∗)				

### 3.3 Charge transfer of molecules

In general, the study of CT in the low-lying excited states of molecules is particularly valuable because these states are more readily accessible in everyday situations. Therefore, we focused primarily on analyzing the CT of the first excited states of dopamine, N,N-dimethyldopamine, and N,N-dihydroxydopamine. In particular only the first excited states of dopamine and N,N-dihydroxydopamine exhibit the CT phenomenon. We also discuss CT images of dopamine and N, N-dihydroxydopamine molecules.

**Fig. 7:**
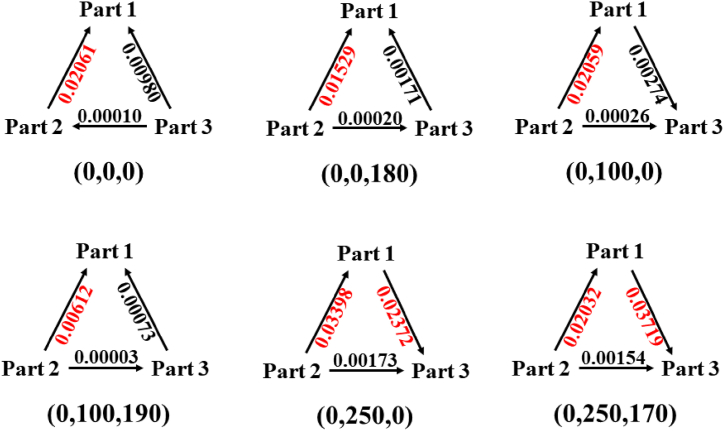
Charge transfer of six conformations of dopamine. Part 1 is phenyl (-C_6_H_3_(OH)_2_), Part 2 is ethyl (-C_2_H_4_), and Part 3 is amino (-NH_2_). The amount of the main transferred charge is represented in red. The calculation method and basis set are CAM-B3LYP/aug-cc-pVTZ.

The CT of the first excited state of the six conformations of dopamine was calculated using the CAM-B3LYP/aug-cc-pVTZ basis set, and the CT was analyzed using Multiwfn 3.7. The summary is listed in Table 5. First, we analyzed the (0,0,0) conformation. Based on the variation in the number, it can be clearly determined that -C_6_H_3_(OH)_2_ (Part 1) obtains electrons, -C_2_H_4_ (Part 2) provides electrons, and (-NH_2_ (Part 1) also provides electrons, but the amount is minimal). To discuss the CT more clearly, we converted the transferred electrons into triangular diagrams, as shown in Fig. 7. It is evident that -NH_2_ transfers electrons to -C_6_H_3_(OH)_2_ and -C_2_H_4_, whereas -C_2_H_4_ transfers electrons to -C_6_H_3_(OH)_2_. In general, -C_2_H_4_ is a donor that provides electrons, and the acceptor is -C_6_H_3_(OH)_2_. Subsequently, we discussed the (0,0,180) and (0,100,190) conformations. The variation in the number indicates that -C_6_H_3_(OH)_2_ obtains electrons, which are provided by -C_2_H_4_ (-NH_2_ also provides a few electrons). As shown in Fig. 7, electrons are transferred from -C_2_H_4_ to -C_6_H_3_(OH)_2_ and -NH_2_, with -NH_2_ transferring a small portion of the electrons to -C_6_H_3_(OH)_2_. Therefore, in the (0,0,180) and (0,100,190) conformations, -C_2_H_4_ remained the donor, whereas -C_6_H_3_(OH)_2_ acted as the acceptor. From the Variation in the number and Fig. 7, -C_2_H_4_ and -C_6_H_3_(OH)_2_ act as donors, whereas -NH_2_ serves as the acceptor. In the (0,250,0) and (0,250,170) conformations, charge transfer from -C_6_H_3_(OH)_2_ to -NH_2_ may occur via spatial interactions, which is consistent with their curled structures.

**Table 5:** Charge transfer of six conformations of dopamine. 1 denotes Part 1, which is phenyl (-C_6_H_3_(OH)_2_). 2 denotes Part 2, which is ethyl (-C_2_H_4_). 3 represents Part 3, which is amino (-NH_2_). Variation of number represents the number of charge changes in the fragment, and transferred electrons represent the amount of electron transfer in the fragment. The calculation method and basis set are CAM-B3LYP/aug-cc-pVTZ.

Conformation	Variation of number			Transferred electrons		
	1	2	3	1 → 2	1 → 3	2 → 3
(0,0,0)	0.03042	−0.02051	−0.00990	−0.02061	−0.00980	−0.00010
(0,0,180)	0.01700	−0.01531	−0.00169	−0.01529	−0.00171	0.00002
(0,100,0)	0.01785	−0.02085	0.00301	−0.02059	0.00274	0.00026
(0,100,190)	0.00685	−0.00615	−0.00070	−0.00612	−0.00073	0.00003
(0,250,0)	0.01026	−0.03571	0.02545	−0.03398	0.02372	0.00173
(0,250,170)	−0.01687	−0.02187	0.03874	−0.02032	0.03719	0.00154

**Fig. 8:**
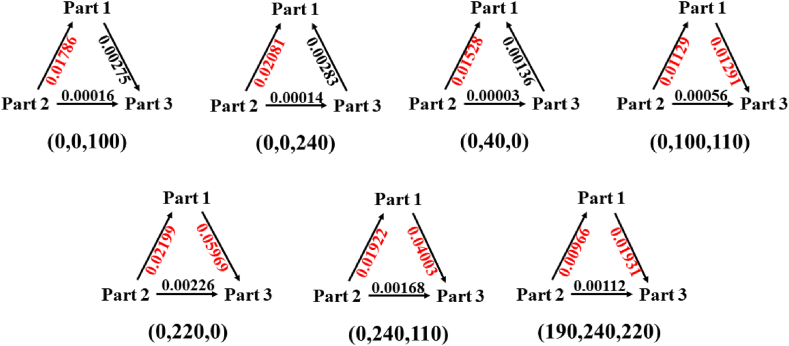
Charge transfer of seven conformations of N,N-dihydroxydopamine. Part 1 is phenyl (-C_6_H_3_(OH)_2_), Part 2 is ethyl (-C_2_H_4_), and Part 3 is amino (-NH_2_(OH)_2_). The amount of the main transferred charge is represented in red. The calculation method and basis set are CAM-B3LYP/aug-cc-pVTZ.

The CT of N and N-dihydroxydopamine was analyzed in a manner similar to that of dopamine. As shown in Table 6 and Fig. 8, these can be divided into two categories. In the first category, which included the (0,0,100), (0,100,110), (0,220,0), (0,240,110) and (190,240,220) conformations, the donors were -C_2_H_4_ and -C_6_H_3_(OH)_2_, and the activators were -NH_2_(OH)_2_ and -C_6_H_3_(OH)_2_. In the second category, in the (0,0,240) and (0,40,0) conformations, the donor is -C_2_H_4_, the acceptor is -NH_2_(OH)_2_ and -C_6_H_3_(OH)_2_ (-NH_2_(OH)_2_ to -C_6_H_3_(OH)_2_ also has some contribution, but the charge is negligible. From the perspective of structure, the more curled the structure, the greater the potential for spatial interaction, facilitating charge transfer through spatial interaction.

**Table 6:** Charge transfer of seven conformations of N,N-dihydroxydopamine. 1 denotes Part 1, which is phenyl (-C_6_H_3_(OH)_2_). 2 represents Part 2, which is ethyl (-C_2_H_4_). 3 represents Part 3, which is amino (-NH_2_(OH)_2_). Variation of number represents the number of charge changes in the fragment, and transferred electrons represent the amount of electron transfer in the fragment. The calculation method and basis set are CAM-B3LYP/aug-cc-pVTZ.

Conformation	Variation of number			Transferred electrons		
	1	2	3	1 → 2	1 → 3	2 → 3
(0,0,100)	0.01511	−0.01802	0.00291	−0.01786	0.00275	0.00016
(0,0,240)	0.02364	−0.02095	−0.00269	−0.02081	−0.00283	0.00014
(0,40,0)	0.01664	−0.01531	−0.00133	−0.01528	−0.00136	0.00003
(0,100,110)	−0.00162	−0.01185	0.01347	−0.01129	0.01291	0.00056
(0,220,0)	−0.03770	−0.02425	0.06195	−0.02199	0.05969	0.00226
(0,240,110)	−0.02081	−0.02090	0.04170	−0.01922	0.04003	0.00168
(190,240,220)	−0.00965	−0.01078	0.02043	−0.00966	0.01931	0.00112

In addition, N,N-dihydroxydopamine exhibited a greater amount of transferred charge, especially in the (0,220,0) and (0,240,110) conformations, where the charge transfer from -C_6_H_3_(OH)_2_ to -NH_2_(OH)_2_ via spatial interactions was more pronounced. Considering that there are more biological molecules in a liquid environment, we speculate that dopamine forms N,N-dihydroxydopamine in organisms, or that dopamine forms hydrogen bonds with water to achieve better signal transmission.

## 4 Conclusions

Different molecular conformations exhibit various properties that can influence their behavior in biological, material, and environmental systems. Understanding the relationship between conformation and CT is crucial to elucidate the mechanisms of molecular activity in organisms. In this study, dopamine, N,N-dimethyldopamine, and N,N-dihydroxydopamine were used as models to explore the relationship between different conformations and CT at the theoretical level, and we hypothesized the mechanism of dopamine activity in organisms. Initially, stable ground-state structures of the molecules were calculated using the MP2/6-311+G(d,p) basis set. Our two-step method examined the conformation of the ionic state and determined that the molecules exhibited nearly equal energy levels and were chiral. The first five excited states of the molecules, excluding chiral symmetry. Interestingly, the first excited states of dopamine and N,N-dihydroxydopamine molecules involve charge transfer excitation, whereas the first excited state of N,N-dimethyldopamine is localized excitation, reflecting the conditions of the human living environment, which typically exists in a low excited state. At the same time, the Rydberg states were distinguished by quantum defects, *HDI* and *EDI* values, and molecular orbitals, noting that the first excited states of the three molecules were the 3s Rydberg states. Finally, the CT of the conformation of dopamine and N,N-dihydroxydopamine in the 3s state was calculated and analyzed, and the curved conformation was more of a spatial interaction. In addition, N,N-dihydroxydopamine has better CT ability than dopamine. It is assumed that in organisms, dopamine forms N,N-dihydroxydopamine, or hydrogen bonds with water to achieve better signal transmission.

## CRediT authorship contribution statement

**Huan An:** Writing – review & editing, Writing – original draft, Validation, Resources, Methodology, Investigation, Formal analysis, Data curation, Conceptualization. **Gulmire Yaermaimaiti:** Visualization, Validation, Formal analysis. **Bumaliya Abulimiti:** Writing – review & editing, Supervision, Methodology, Funding acquisition, Data curation. **Mei Xiang:** Writing – review & editing, Resources, Project administration, Funding acquisition, Data curation. **Xiaoning Wang:** Writing – review & editing, Validation, Supervision, Methodology, Data curation.

## Data availability statement

The data are included in article and supplementary materials in this article.

## Declaration of competing interest

The authors declare that they have no known competing financial interests or personal relationships that could have appeared to influence the work reported in this paper.

## Footnotes

Appendix A Supplementary data to this article can be found online at https://doi.org/10.1016/j.heliyon.2025.e42058.
