# Supplementary material for: Theoretical study on the conformation-dependent charge transfer of the excited state of dopamine
Source: Heliyon. 2025 Jan 17;11(2):e42058. doi: 10.1016/j.heliyon.2025.e42058 (PMC11791279; doi:10.1016/j.heliyon.2025.e42058)
Supplement: Multimedia component 1 [file mmc1.docx]

**Supplementary Material for**

**“Theoretical study on the conformation-dependent charge transfer of the excited state of dopamine”**

Huan An^a,b^, Asiya^a^, Gulmire Yaermaimaiti^a^, Bumaliya Abulimiti^a,b^, Mei Xiang^a^, Xiaoning Wang^a^

^a^ Xinjiang Key Laboratory for Luminescence Minerals and Optical Functional Materials, School of Physics and Electronic Engineering, Xinjiang Normal University, Urumqi 830054, China.

^b^ School of Chemistry and Chemical Engineering, Xinjiang Normal University, Urumqi 830054, China.

**Correspondence**

Bumaliya Abulimiti, School of Physics and Electronic Engineering, Xinjiang Normal University, Urumqi 830054, China. Emile: maryam917@163.com.

Mei Xiang, School of Physics and Electronic Engineering, Xinjiang Normal University, Urumqi 830054, China. Emile: mei811014@126.com.

**Contents:**

[S1. The ground state types of dopamine 2](#_Toc172117832)

[S2. The Boltzmann distribution of ion state at different vibrational temperatures 3](#_Toc172117833)

[S3. The excited state types of dopamine 4](#_Toc172117834)

[S4. The excited state types of N,N-dimethyldopamine 5](#_Toc172117835)

[S5. Excited state types of N,N-dihydroxydopamine 6](#_Toc172117836)

[S6. Excited state information of dopamine 7](#_Toc172117837)

[S7. Excited state information of N,N-dimethyldopamine 10](#_Toc172117838)

[S8. Excited state information of N, N-dihydroxydopamine 12](#_Toc172117839)

[S9. References 15](#_Toc172117840)

# **S1. The ground state types of dopamine**

Table S1. The ground state structure parameters of dopamine. The calculation method and basis set is: MP2/6-311+G(d,p).

| Parameters^a^ | Calculated | EXP.^b^ | EXP.^c^ | Parameters^a^ | Calculated | EXP.^b^ | EXP.^c^ |
| --- | --- | --- | --- | --- | --- | --- | --- |
| C1-C2 | 1.39365 | 1.392 | 1.381 | C1-C2-C3 | 120.55181 | 120.760 | 120.400 |
| C2-C3 | 1.40544 | 1.404 | 1.385 | C2-C3-C4 | 118.35122 | 118.050 | 119.100 |
| C3-C4 | 1.40257 | 1.401 | 1.393 | C3-C4-C5 | 121.18528 | 121.290 | 120.900 |
| C4-C5 | 1.40003 | 1.397 | 1.385 | C4-C5-C6 | 120.03752 | 120.170 | 119.800 |
| C5-C6 | 1.39483 | 1.392 | 1.385 | C5-C6-C1 | 119.17596 | 119.050 | — |
| C6-C1 | 1.40439 | 1.402 | 1.396 | C6-C1-C2 | 120.66585 | 120.640 | 120.400 |
| C1-O12 | 1.38093 | 1.381 | 1.364 | C6-C1-O12 | 114.95779 | 115.210 | 121.600 |
| C2-H7 | 1.09025 | — | — | C2-C1-O12 | 124.35328 | 124.130 | 118.000 |
| C3-C14 | 1.50687 | 1.515 | 1.497 | C1-C6-O10 | 120.75269 | 120.890 | 116.900 |
| C4-H8 | 1.08787 | — | — | C5-C6-O10 | 120.05947 | 120.050 | 123.500 |
| C5-H9 | 1.08635 | — | — | C2-C3-C14 | 120.04635 | 120.590 | 120.500 |
| C6-O10 | 1.36363 | 1.367 | 1.365 | C4-C3-C14 | 121.43739 | 121.320 | 120.500 |
| O10-H11 | 0.96612 | 0.969 | 0.840 | C3-C14-C17 | 111.47455 | 113.730 | 111.000 |
| O12-H13 | 0.96224 | 0.965 | 0.900 | C14-C17-N20 | 110.10706 | 111.170 | — |
| C14-H15 | 1.09591 | 1.096 | 1.030 | C17-N20-H21 | 110.39471 | 110.840 | 116.000 |
| C14-H16 | 1.09774 | 1.095 | 0.860 | C17-N20-H22 | 109.77853 | 110.480 | 115.000 |
| C14-C17 | 1.53142 | 1.540 | 1.511 | H21-N20-H22 | 106.63754 | 107.200 | 122.000 |
| C17-H18 | 1.10130 | — | — | C1-C6-O10-H11 | -3.59159 | 0.240 | — |
| C17-H19 | 1.09553 | — | — | H19-C17-N20-H21 | -58.08117 | -54.620 | — |
| C17-N20 | 1.46651 | 1.467 | 1.479 | H19-C17-N20-H22 | -175.35929 | -173.310 | — |
| N20-H21 | 1.01572 | 1.017 | 0.930 | C2-C3-C14-C17 | -81.61693 | — | — |
| N20-H22 | 1.01623 | 1.016 | 0.930 | C14-C17-N20-H22 | 65.81508 | 68.020 | — |

^a^ The unit of bond lengths are Å, and the angles are degree (º).

^b^ Taken from Ref. [1].

^c^ Taken from Ref. [2].

# **S2. The Boltzmann distribution of ion state at different vibrational temperatures**

Table S2. The Boltzmann distribution of the six conformations of dopamine ion state at different vibrational temperatures.

| Temperature (K) | (0,0,0) | (0,0,180) | (0,100,0) | (0,100,190) | (0,250,0) | (0,250,170) |
| --- | --- | --- | --- | --- | --- | --- |
| 273.15 | 81.37 % | 3.20 % | 0.74 % | 1.85 % | 2.05 % | 10.79 % |
| 283.15 | 79.88 % | 3.53 % | 0.86 % | 2.08 % | 2.29 % | 11.37 % |
| 293.15 | 78.39 % | 3.85 % | 0.98 % | 2.31 % | 2.54 % | 11.93 % |
| 298.15 | 77.66 % | 4.01 % | 1.05 % | 2.43 % | 2.66 % | 12.20 % |
| 303.15 | 76.92 % | 4.17 % | 1.11 % | 2.54 % | 2.79 % | 12.46 % |
| 323.15 | 74.05 % | 4.81 % | 1.39 % | 3.02 % | 3.30 % | 13.42 % |
| 373.15 | 67.39 % | 6.32 % | 2.16 % | 4.22 % | 4.55 % | 15.36 % |
| 473.15 | 56.68 % | 8.76 % | 3.76 % | 6.38 % | 6.77 % | 17.65 % |
| 773.15 | 39.47 % | 12.59 % | 7.50 % | 10.37 % | 10.75 % | 19.33 % |
| 1273.15 | 29.32 % | 14.65 % | 10.70 % | 13.02 % | 13.31 % | 19.02 % |

Table S3. The Boltzmann distribution of the six conformations of N,N-dimethyldopamine ion state at different vibrational temperatures.

| Temperature (K) | (0,0,0) | (0,0,180) | (0,100,10) | (0,260,340) |
| --- | --- | --- | --- | --- |
| 273.15 | 98.40 % | 1.23 % | 0.35 % | 0.02 % |
| 283.15 | 98.12 % | 1.43 % | 0.43 % | 0.02 % |
| 293.15 | 97.81 % | 1.65 % | 0.51 % | 0.03 % |
| 298.15 | 97.64 % | 1.76 % | 0.56 % | 0.04 % |
| 303.15 | 97.47 % | 1.88 % | 0.61 % | 0.04 % |
| 323.15 | 96.73 % | 2.38 % | 0.83 % | 0.07 % |
| 373.15 | 94.48 % | 3.82 % | 1.53 % | 0.18 % |
| 473.15 | 88.87 % | 7.07 % | 3.44 % | 0.62 % |
| 773.15 | 71.57 % | 15.21 % | 9.78 % | 3.44 % |
| 1273.15 | 54.13 % | 21.13 % | 16.17 % | 8.57 % |

Table S4. The Boltzmann distribution of the six conformations of N,N-dihydroxydopamine ion state at different vibrational temperatures.

| Temperature (K) | (0,0,100) | (0,0,240) | (0,40,0) | (0,100,110) | (0,220,0) | (0,240,100) | (190,240,220) |
| --- | --- | --- | --- | --- | --- | --- | --- |
| 273.15 | 0.00 % | 30.13 % | 0.11 % | 0.00 % | 0.02 % | 0.00 % | 69.73 % |
| 283.15 | 0.01 % | 30.75 % | 0.14 % | 0.00 % | 0.03 % | 0.00 % | 69.08 % |
| 293.15 | 0.01 % | 31.32 % | 0.17 % | 0.01 % | 0.04 % | 0.00 % | 68.46 % |
| 298.15 | 0.01 % | 31.60 % | 0.18 % | 0.01 % | 0.04 % | 0.00 % | 68.16 % |
| 303.15 | 0.01 % | 31.86 % | 0.20 % | 0.01 % | 0.05 % | 0.00 % | 67.86 % |
| 323.15 | 0.02 % | 32.85 % | 0.28 % | 0.01 % | 0.07 % | 0.01 % | 66.76 % |
| 373.15 | 0.06 % | 34.81 % | 0.57 % | 0.04 % | 0.17 % | 0.02 % | 64.33 % |
| 473.15 | 0.24 % | 37.15 % | 1.45 % | 0.17 % | 0.56 % | 0.11 % | 60.31 % |
| 773.15 | 1.73 % | 37.44 % | 5.15 % | 1.38 % | 2.88 % | 1.07 % | 50.36 % |
| 1273.15 | 4.96 % | 32.13 % | 9.63 % | 4.33 % | 6.77 % | 3.70 % | 38.47 % |

# **S3. The excited state types of dopamine**

Table S5. The excited state types of 6 conformations of dopamine. CT is charging transfer excitation and LE is local excitation. The calculation method and basis set are: CAM-B3LYP/aug-cc-pVTZ.

| Conformation |  | *Sr* | *D* | *H* | *σ_h_* | *σ_e_* | *t* | *HDI* | *EDI* | E (eV) | *δ* | States | Transition |
| --- | --- | --- | --- | --- | --- | --- | --- | --- | --- | --- | --- | --- | --- |
| (0,0,0) | S_0_→S_1_ | 0.27781 | 2.691 | 2.622 | 2.132 | 3.111 | 1.208 | 9.61 | 3.93 | 4.481 | 0.993 | 3s | CT(π→n*) |
|  | S_0_→S_2_ | 0.81276 | 0.279 | 0.263 | 2.152 | 2.375 | -1.187 | 8.69 | 7.31 | 4.676 |  |  | LE(π→δ*) |
|  | S_0_→S_3_ | 0.44131 | 2.021 | 3.542 | 2.844 | 4.240 | -0.583 | 8.46 | 2.27 | 5.084 | 0.785 | 3p_x_ | CT(π→σ*) |
|  | S_0_→S_4_ | 0.28318 | 0.558 | 3.389 | 2.250 | 4.528 | -1.731 | 9.22 | 1.77 | 5.219 | 0.729 | 3p_y_ | LE(π→π*) |
|  | S_0_→S_5_ | 0.76897 | 0.821 | 2.949 | 2.604 | 3.294 | -1.458 | 8.26 | 6.10 | 5.285 |  |  | LE(π→π*) |
| (0,0,180) | S_0_→S_1_ | 0.28192 | 2.634 | 2.572 | 2.048 | 3.097 | 1.161 | 9.74 | 3.41 | 4.459 | 0.999 | 3s | CT(π→n*) |
|  | S_0_→S_2_ | 0.81777 | 0.330 | 2.214 | 2.077 | 2.351 | -1.117 | 7.78 | 7.31 | 4.694 |  |  | LE(π→δ*) |
|  | S_0_→S_3_ | 0.38722 | 1.383 | 3.257 | 2.135 | 4.378 | -0.3 | 9.41 | 2.26 | 5.191 | 0.741 | 3p_x_ | LE(π→π*) |
|  | S_0_→S_4_ | 0.51516 | 1.165 | 3.100 | 2.159 | 4.041 | -0.714 | 9.12 | 2.68 | 5.249 | 0.716 | 3p_y_ | LE(π→π*) |
|  | S_0_→S_5_ | 0.69436 | 1.427 | 2.919 | 2.241 | 3.597 | -0.594 | 8.84 | 4.77 | 5.316 |  |  | LE(π→π*) |
| (0,100,0) | S_0_→S_1_ | 0.28739 | 2.594 | 2.607 | 2.072 | 3.143 | 1.092 | 9.63 | 3.31 | 4.516 | 0.982 | 3s | CT(π→n*) |
|  | S_0_→S_2_ | 0.81382 | 0.330 | 2.249 | 2.101 | 2.397 | -1.114 | 8.71 | 7.23 | 4.673 |  |  | LE(π→δ*) |
|  | S_0_→S_3_ | 0.55484 | 1.840 | 3.133 | 2.392 | 3.874 | -0.271 | 8.31 | 2.99 | 5.154 | 0.757 | 3p_x_ | LE(π→π*) |
|  | S_0_→S_4_ | 0.44978 | 0.512 | 3.223 | 2.190 | 4.256 | -0.929 | 9.05 | 2.16 | 5.220 | 0.729 | 3p_y_ | LE(π→π*) |
|  | S_0_→S_5_ | 0.62724 | 0.472 | 3.065 | 2.261 | 3.870 | -0.865 | 8.77 | 3.92 | 5.363 |  |  | LE(π→π*) |
| (0,100,190) | S_0_→S_1_ | 0.27505 | 2.673 | 2.592 | 2.036 | 3.148 | 1.188 | 9.78 | 3.46 | 4.348 | 1.031 | 3s | CT(π→n*) |
|  | S_0_→S_2_ | 0.80443 | 0.449 | 2.273 | 2.068 | 2.479 | -1.019 | 8.86 | 7.03 | 4.676 |  |  | LE(π→δ*) |
|  | S_0_→S_3_ | 0.42582 | 2.492 | 3.212 | 2.098 | 4.325 | 0.186 | 9.28 | 2.11 | 5.014 | 0.813 | 3p_x_ | CT(π→n*) |
|  | S_0_→S_4_ | 0.24915 | 0.880 | 3.289 | 2.061 | 4.517 | -1.179 | 9.63 | 2.23 | 5.103 | 0.778 | 3p_y_ | LE(π→π*) |
|  | S_0_→S_5_ | 0.65384 | 1.840 | 2.945 | 2.166 | 3.725 | -0.114 | 8.91 | 4.12 | 5.322 | 0.684 | 3p_z_ | LE(π→π*) |
| (0,250,0) | S_0_→S_1_ | 0.28757 | 2.613 | 2.641 | 2.124 | 3.158 | 1.158 | 9.55 | 3.35 | 4.445 | 1.003 | 3s | CT(π→n*) |
|  | S_0_→S_2_ | 0.81080 | 0.347 | 2.263 | 2.123 | 2.403 | -1.117 | 8.73 | 7.24 | 4.669 |  |  | LE(π→δ*) |
|  | S_0_→S_3_ | 0.59254 | 1.447 | 3.211 | 2.413 | 4.009 | -0.746 | 8.40 | 3.42 | 5.105 | 0.777 | 3p_x_ | LE(π→π*) |
|  | S_0_→S_4_ | 0.32336 | 0.442 | 3.308 | 2.177 | 4.438 | -1.697 | 9.23 | 1.79 | 5.205 | 0.735 | 3p_y_ | LE(π→σ*) |
|  | S_0_→S_5_ | 0.64661 | 0.892 | 3.038 | 2.468 | 3.608 | -0.871 | 8.27 | 4.11 | 5.296 |  |  | LE(π→π*) |
| (0,250,170) | S_0_→S_1_ | 0.29446 | 2.569 | 2.655 | 2.056 | 3.253 | 1.083 | 9.68 | 3.23 | 4.458 | 1.000 | 3s | CT(π→n*) |
|  | S_0_→S_2_ | 0.81286 | 0.409 | 2.307 | 2.090 | 2.523 | -1.110 | 8.71 | 6.98 | 4.724 |  |  | LE(π→δ*) |
|  | S_0_→S_3_ | 0.42473 | 2.550 | 3.280 | 2.193 | 4.367 | 0.238 | 8.93 | 2.07 | 5.063 | 0.794 | 3p_x_ | CT(π→σ*) |
|  | S_0_→S_4_ | 0.32517 | 0.477 | 3.356 | 2.111 | 4.600 | -1.352 | 9.34 | 1.72 | 5.147 | 0.760 | 3p_y_ | LE(π→π*) |
|  | S_0_→S_5_ | 0.68671 | 0.995 | 2.909 | 2.144 | 3.675 | -0.836 | 8.92 | 4.69 | 5.317 |  |  | LE(π→π*) |

# S4. The excited state types of N,N-dimethyldopamine

Table S6. The excited state types of 4 conformations of N,N-dimethyldopamine. CT is charging transfer excitation and LE is local excitation. The calculation method and basis set are: CAM-B3LYP/aug-cc-pVTZ.

| Conformation |  | *Sr* | *D* | *H* | *σ_h_* | *σ_e_* | *t* | *HDI* | *EDI* | E (eV) | *δ* | States | Transition |
| --- | --- | --- | --- | --- | --- | --- | --- | --- | --- | --- | --- | --- | --- |
| (0,0,0) | S_0_→S_1_ | 0.29325 | 0.715 | 2.773 | 1.742 | 3.803 | -0.934 | 18.54 | 2.10 | 4.309 | 0.913 | 3s | LE(σ→σ*) |
|  | S_0_→S_2_ | 0.22423 | 1.884 | 3.667 | 2.583 | 4.751 | -0.429 | 15.85 | 1.86 | 4.873 | 0.694 | 3p_y_ | LE(σ→σ*) |
|  | S_0_→S_3_ | 0.26015 | 0.792 | 3.202 | 1.094 | 4.310 | -1.144 | 17.62 | 1.78 | 4.965 | 0.651 | 3p_z_ | LE(σ→σ*) |
|  | S_0_→S_4_ | 0.32570 | 1.276 | 4.114 | 3.294 | 4.935 | -1.207 | 10.11 | 1.97 | 5.023 |  |  | LE(σ→σ*)  LE(π→π*) |
|  | S_0_→S_5_ | 0.37588 | 0.607 | 3.935 | 3.078 | 4.793 | -2.410 | 13.06 | 2.15 | 5.154 | 0.556 | 3p_x_ | LE(σ→σ*)  LE(π→δ*) |
| (0,0,180) | S_0_→S_1_ | 0.29581 | 0.745 | 2.874 | 1.896 | 3.853 | -1.043 | 18.04 | 2.03 | 4.335 | 0.904 | 3s | CT(π→n*) |
|  | S_0_→S_2_ | 0.64404 | 1.241 | 2.996 | 2.810 | 3.183 | -0.548 | 8.25 | 5.15 | 4.732 | 0.755 | 3p_x_ | LE(n→δ*)  LE(π→δ*) |
|  | S_0_→S_3_ | 0.46377 | 1.692 | 3.727 | 3.234 | 4.221 | -0.637 | 9.63 | 2.96 | 4.857 | 0.701 | 3p_y_ | LE(n→δ*)  LE(π→δ*) |
|  | S_0_→S_4_ | 0.28211 | 0.907 | 3.985 | 2.968 | 5.001 | -1.704 | 13.97 | 1.58 | 4.960 |  |  | LE(σ→σ*)  LE(π→π*) |
|  | S_0_→S_5_ | 0.28997 | 0.602 | 3.464 | 2.493 | 4.435 | -1.407 | 16.11 | 1.78 | 4.988 | 0.640 | 3p_z_ | LE(σ→σ*)  LE(π→π*) |
| (0,100,100) | S_0_→S_1_ | 0.29054 | 0.963 | 2.818 | 1.643 | 3.994 | -0.677 | 18.24 | 1.97 | 4.371 | 0.891 | 3s | LE(σ→σ*) |
|  | S_0_→S_2_ | 0.43453 | 2.494 | 2.580 | 2.230 | 2.930 | 0.828 | 14.58 | 6.35 | 4.637 | 0.793 | 3p_x_ | CT(σ→δ*) |
|  | S_0_→S_3_ | 0.63502 | 0.954 | 3.101 | 2.683 | 3.519 | -0.914 | 9.06 | 4.53 | 4.813 |  |  | LE(σ→π*)  LE(π→π*) |
|  | S_0_→S_4_ | 0.28945 | 1.599 | 3.361 | 2.099 | 4.622 | -0.504 | 16.11 | 1.88 | 4.850 | 0.704 | 3p_y_ | LE (σ→π*) |
|  | S_0_→S_5_ | 0.38709 | 1.181 | 3.452 | 2.438 | 4.467 | -0.913 | 14.16 | 2.75 | 4.929 | 0.668 | 3p_z_ | LE(σ→π*)  LE(π→π*) |
| (0,260,340) | S_0_→S_1_ | 0.28674 | 0.941 | 2.838 | 1.669 | 4.008 | -0.692 | 18.24 | 1.92 | 4.338 | 0.903 | 3s | LE(σ→σ*) |
|  | S_0_→S_2_ | 0.48333 | 2.125 | 2.734 | 2.426 | 3.043 | 0.284 | 13.30 | 6.10 | 4.612 |  |  | CT(σ→δ*) LE(π→δ*) |
|  | S_0_→S_3_ | 0.29221 | 1.915 | 3.362 | 2.165 | 4.559 | -0.085 | 15.71 | 2.08 | 4.787 | 0.732 | 3p_y_ | CT(π→π*) |
|  | S_0_→S_4_ | 0.49799 | 0.489 | 3.370 | 2.620 | 4.119 | -1.392 | 11.59 | 3.19 | 4.859 | 0.700 | 3p_z_ | LE(σ→π*)  LE(π→π*) |
|  | S_0_→S_5_ | 0.49447 | 2.039 | 3.125 | 2.640 | 3.663 | -0.069 | 12.11 | 4.54 | 4.902 | 0.653 | 3p_x_ | CT(σ→δ*) LE(π→δ*) |

# S5. Excited state types of N,N-dihydroxydopamine

Table S7. Excited state types of seven conformations of N,N-dihydroxydopamine. CT is charging transfer excitation and LE is local excitation. The calculation method and basis set are: CAM-B3LYP/aug-cc-pVTZ.

| Conformation |  | *Sr* | *D* | *H* | *σ_h_* | *σ_e_* | *t* | *HDI* | *EDI* | E (eV) | *δ* | States | Transition |
| --- | --- | --- | --- | --- | --- | --- | --- | --- | --- | --- | --- | --- | --- |
| (0,0,0) | S_0_→S_1_ | 0.29133 | 2.570 | 2.571 | 2.029 | 3.114 | 1.093 | 9.78 | 3.41 | 4.530 | 0.957 | 3s | CT(π→n*) |
|  | S_0_→S_2_ | 0.82177 | 0.361 | 2.213 | 2.076 | 2.350 | -1.055 | 8.81 | 7.36 | 4.708 |  |  | LE(π→δ*) |
|  | S_0_→S_3_ | 0.69108 | 1.671 | 2.777 | 2.107 | 3.448 | -0.051 | 9.12 | 4.92 | 5.258 | 0.682 | 3p_z_ | LE(π→π*) |
|  | S_0_→S_4_ | 0.32417 | 1.013 | 3.159 | 2.061 | 4.256 | -0.931 | 9.58 | 2.06 | 5.290 | 0.667 | 3p_y_ | LE(π→π*) |
|  | S_0_→S_5_ | 0.57825 | 0.964 | 3.105 | 2.097 | 4.113 | -1.230 | 9.26 | 3.42 | 5.400 | 0.614 | 3p_x_ | LE(π→π*) |
| (0,0,180) | S_0_→S_1_ | 0.28517 | 2.639 | 2.619 | 2.095 | 3.143 | 1.142 | 9.65 | 3.28 | 4.474 | 0.975 | 3s | CT(π→n*) |
|  | S_0_→S_2_ | 0.81970 | 0.301 | 2.227 | 2.113 | 2.340 | -1.175 | 8.71 | 7.37 | 4.688 |  |  | LE(π→δ*) |
|  | S_0_→S_3_ | 0.27276 | 1.325 | 3.638 | 2.406 | 4.870 | -1.004 | 8.89 | 1.60 | 5.222 | 0.698 | 3p_y_ | LE(π→π*) |
|  | S_0_→S_4_ | 0.25028 | 1.496 | 3.881 | 2.499 | 5.263 | -0.805 | 8.85 | 1.85 | 5.239 | 0.691 | 3p_x_ | LE(π→π*) |
|  | S_0_→S_5_ | 0.83146 | 0.771 | 2.494 | 2.240 | 2.748 | -0.947 | 8.77 | 7.33 | 5.277 | 0.673 | 3p_z_ | LE(π→π*) |
| (0,100,0) | S_0_→S_1_ | 0.28052 | 2.665 | 2.565 | 2.037 | 3.092 | 1.127 | 9.77 | 3.49 | 4.400 | 0.997 | 3s | CT(π→n*) |
|  | S_0_→S_2_ | 0.81399 | 0.369 | 2.224 | 2.080 | 2.369 | -1.071 | 8.81 | 7.25 | 4.691 |  |  | LE(π→δ*) |
|  | S_0_→S_3_ | 0.53469 | 1.942 | 3.187 | 2.116 | 4.267 | -0.300 | 9.27 | 3.02 | 5.110 | 0.747 | 3p_z_ | CT(π→π*)  CT(n→π*) |
|  | S_0_→S_4_ | 0.35949 | 0.761 | 3.226 | 2.080 | 4.371 | -1.378 | 9.56 | 2.01 | 5.168 | 0.722 | 3p_y_ | LE(π→π*)  LE(n→π*) |
|  | S_0_→S_5_ | 0.69439 | 1.224 | 2.918 | 2.126 | 3.711 | -0.903 | 9.11 | 4.76 | 5.360 | 0.634 | 3p_x_ | LE(π→π*) LE(n→π*) |
| (0,100,190) | S_0_→S_1_ | 0.27915 | 2.622 | 2.609 | 2.044 | 3.174 | 1.138 | 9.70 | 3.39 | 4.442 | 0.984 | 3s | CT(π→n*) |
|  | S_0_→S_2_ | 0.80292 | 0.386 | 2.261 | 2.074 | 2.449 | -0.961 | 8.85 | 7.04 | 4.665 |  |  | LE(π→δ*) |
|  | S_0_→S_3_ | 0.30339 | 2.595 | 3.125 | 2.157 | 4.094 | 0.731 | 9.19 | 2.29 | 5.106 | 0.749 | 3p_y_ | CT(π→σ*)  CT(σ→σ*) |
|  | S_0_→S_4_ | 0.33192 | 1.248 | 3.334 | 2.100 | 4.568 | -0.480 | 9.44 | 2.18 | 5.198 | 0.709 | 3p_x_ | LE(π→π*) |
|  | S_0_→S_5_ | 0.70743 | 0.694 | 2.823 | 2.109 | 3.536 | -0.933 | 9.01 | 4.88 | 5.286 | 0.669 | 3p_z_ | LE(π→π*) |
| (0,250,0) | S_0_→S_1_ | 0.28008 | 2.595 | 2.645 | 2.054 | 3.236 | 1.147 | 9.72 | 3.29 | 4.423 | 0.990 | 3s | CT(π→n*) |
|  | S_0_→S_2_ | 0.80359 | 0.368 | 2.291 | 2.073 | 2.508 | -1.042 | 8.82 | 7.00 | 4.696 |  |  | LE(π→δ*) |
|  | S_0_→S_3_ | 0.37802 | 1.851 | 3.237 | 2.129 | 4.345 | -0.112 | 9.24 | 1.90 | 5.143 | 0.733 | 3p_y_ | LE(π→δ*) |
|  | S_0_→S_4_ | 0.64750 | 1.547 | 2.968 | 2.171 | 3.766 | -0.387 | 9.03 | 4.12 | 5.171 | 0.721 | 3p_x_ | LE(π→σ*) |
|  | S_0_→S_5_ | 0.32669 | 1.492 | 3.203 | 2.084 | 4.322 | -0.321 | 9.56 | 2.19 | 5.196 | 0.710 | 3p_z_ | LE(π→σ*) |
| (0,250,170) | S_0_→S_1_ | 0.27154 | 2.670 | 2.641 | 2.048 | 3.234 | 1.215 | 9.70 | 3.19 | 4.345 | 1.013 | 3s | CT(π→n*) |
|  | S_0_→S_2_ | 0.80293 | 0.337 | 2.266 | 2.080 | 2.452 | -1.015 | 8.84 | 7.06 | 4.657 |  |  | LE(π→δ*) |
|  | S_0_→S_3_ | 0.50654 | 1.950 | 3.195 | 2.112 | 4.278 | -0.147 | 9.30 | 2.70 | 5.028 | 0.781 | 3p_x_ | CT(π→σ*) |
|  | S_0_→S_4_ | 0.32900 | 0.861 | 3.274 | 2.078 | 4.469 | -1.184 | 9.58 | 1.91 | 5.111 | 0.747 | 3p_y_ | LE(π→σ*) |
|  | S_0_→S_5_ | 0.41969 | 2.850 | 3.041 | 2.102 | 3.980 | 0.995 | 9.30 | 2.64 | 5.182 | 0.716 | 3p_z_ | CT(π→n*) |
| (0,250,170) | S_0_→S_1_ | 0.27065 | 2.666 | 2.723 | 2.120 | 3.325 | 1.125 | 9.46 | 3.17 | 4.296 | 1.027 | 3s | CT(π→n*) |
|  | S_0_→S_2_ | 0.78542 | 0.422 | 2.344 | 2.140 | 2.547 | -0.983 | 8.73 | 6.80 | 4.607 |  |  | LE(π→δ*) |
|  | S_0_→S_3_ | 0.26931 | 3.032 | 3.375 | 2.416 | 4.334 | 0.756 | 8.56 | 2.16 | 4.835 | 0.854 | 3p_x_ | CT(π→n*)  CT(σ→n*) |
|  | S_0_→S_4_ | 0.35113 | 1.193 | 3.334 | 2.186 | 4.481 | -0.637 | 9.20 | 1.98 | 5.004 | 0.790 | 3p_y_ | LE(π→σ*) |
|  | S_0_→S_5_ | 0.57657 | 0.936 | 3.189 | 2.245 | 4.133 | -1.144 | 8.80 | 3.16 | 5.153 | 0.729 | 3p_z_ | LE(π→π*) |

# S6. Excited state information of dopamine


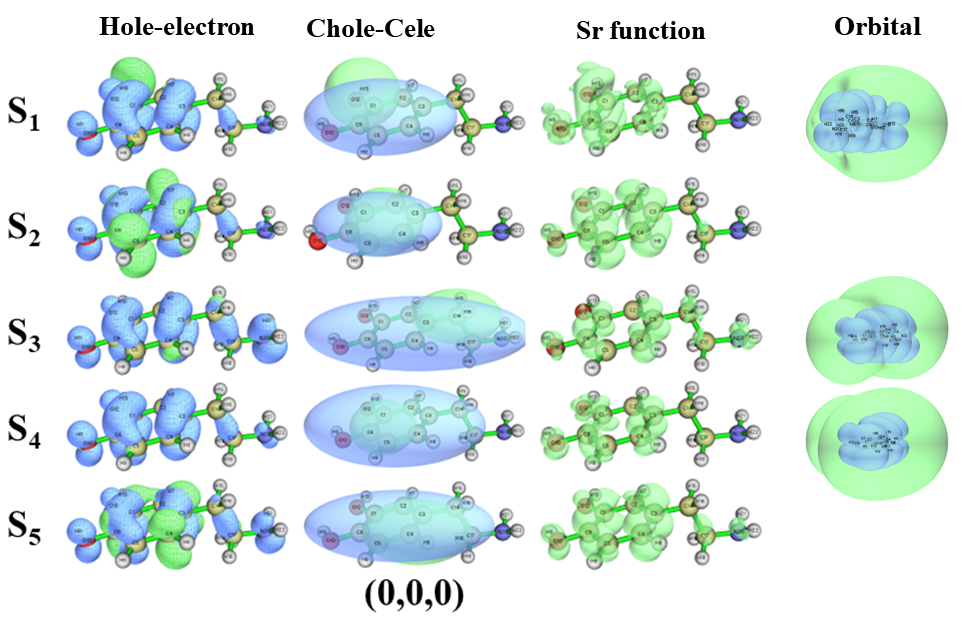


Fig. S1. The hole-electron diagram, Chole-Cele diagram, Sr function diagram and molecular orbital of the five excited states of dopamine (0,0,0) conformation. The calculation method and basis set are: CAM-B3LYP/aug-cc-pVTZ.


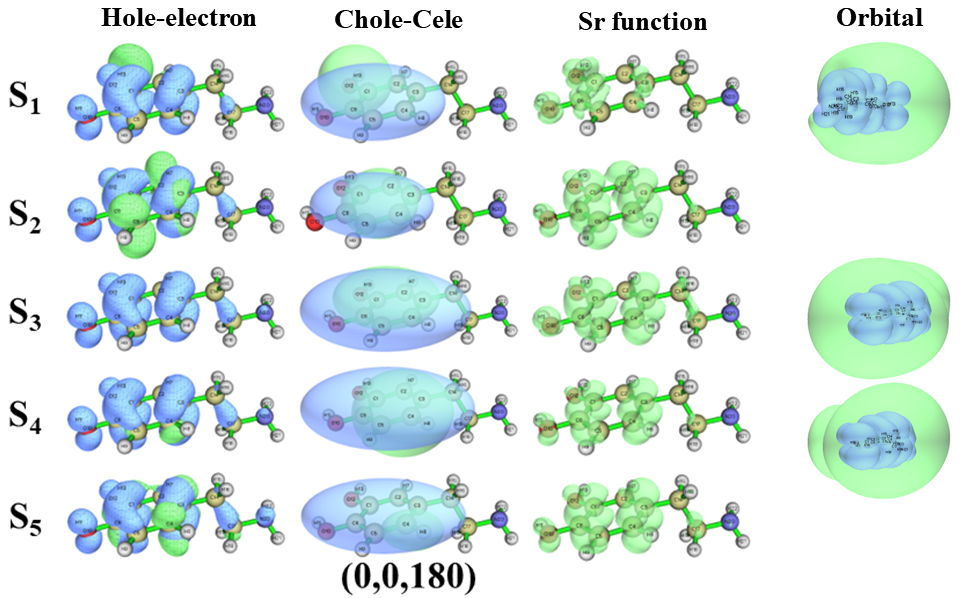


Fig. S2. The hole-electron diagram, Chole-Cele diagram, Sr function diagram and molecular orbital of the five excited states of the dopamine (0,0,180) conformation. The calculation method and basis set are: CAM-B3LYP/aug-cc-pVTZ.


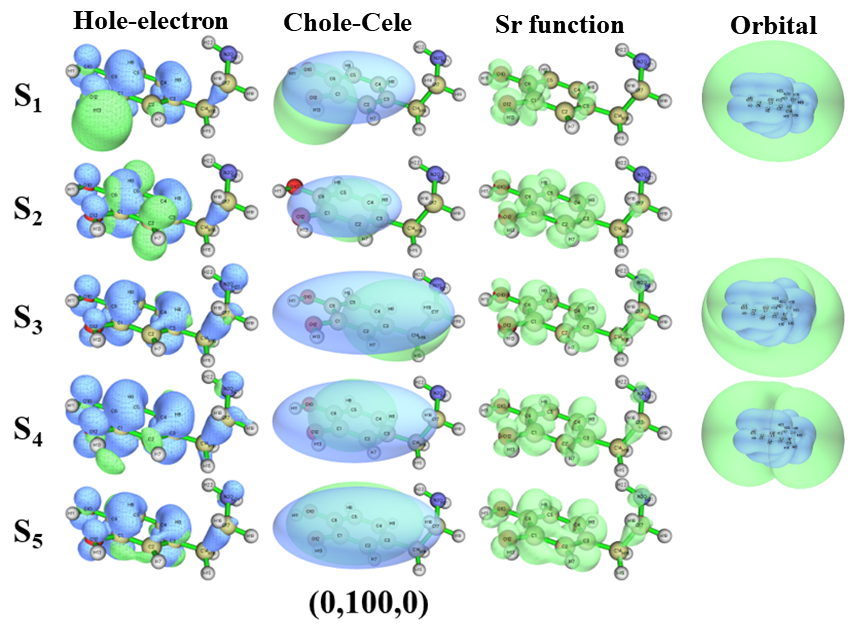


Fig. S3. The hole-electron diagram, Chole-Cele diagram, Sr function diagram and molecular orbital of the five excited states of dopamine (0,100,0) conformation. The calculation method and basis set are: CAM-B3LYP/aug-cc-pVTZ.


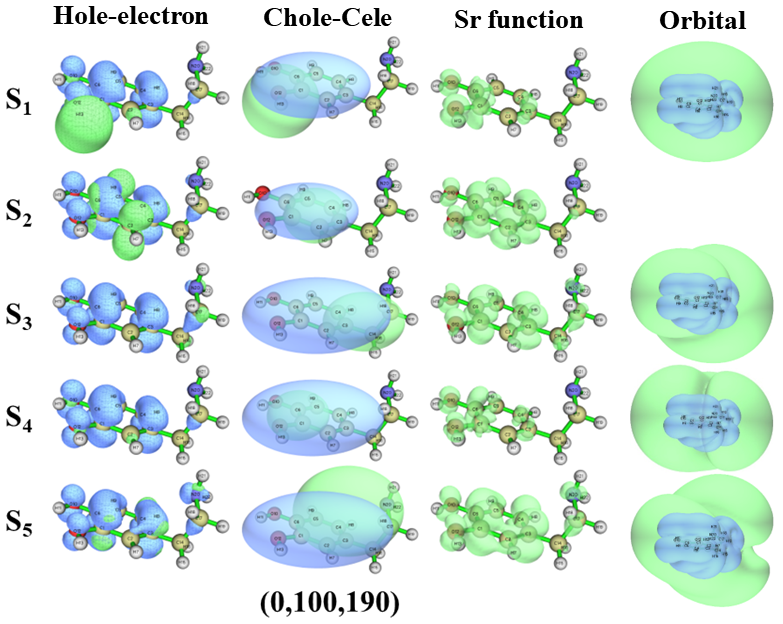


Fig. S4. The hole-electron diagram, Chole-Cele diagram, Sr function diagram and molecular orbital of the five excited states of dopamine (0,100,190) conformation. The calculation method and basis set are: CAM-B3LYP/aug-cc-pVTZ.


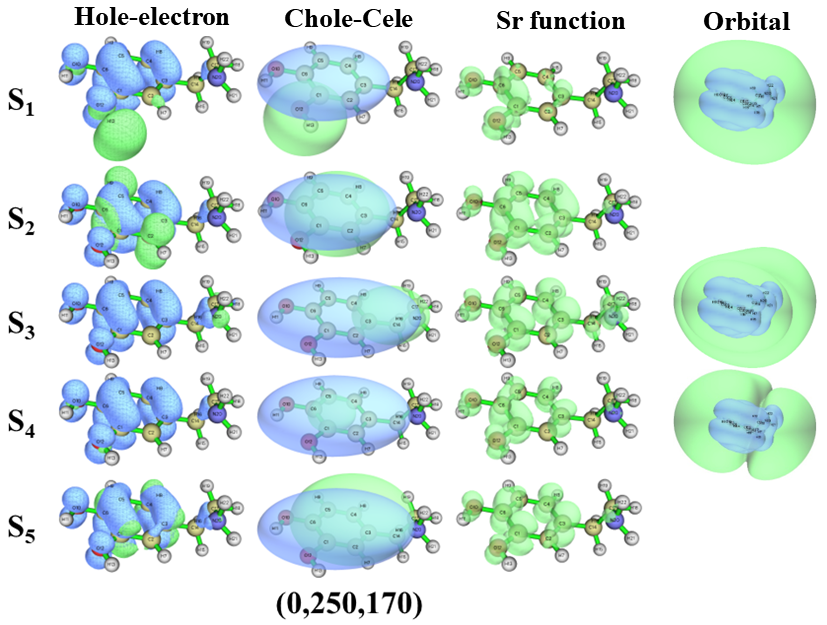


Fig. S5. The hole-electron diagram, Chole-Cele diagram, Sr function diagram and molecular orbital of the five excited states of dopamine (0,250,170) conformation. The calculation method and basis set are: CAM-B3LYP/aug-cc-pVTZ.


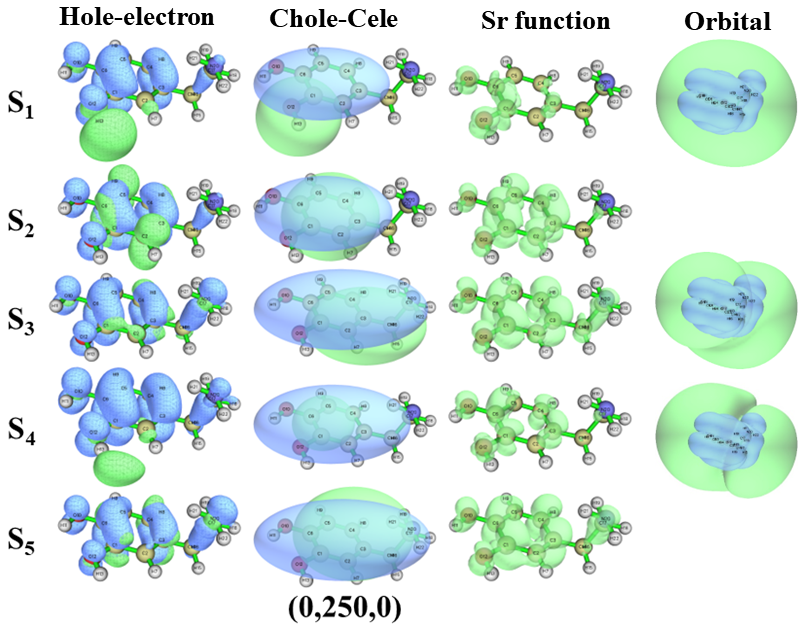


Fig. S6. The hole-electron diagram, Chole-Cele diagram, Sr function diagram and molecular orbital of the five excited states of dopamine (0,250,0) conformation. The calculation method and basis set are: CAM-B3LYP/aug-cc-pVTZ.

# S7. Excited state information of N,N-dimethyldopamine


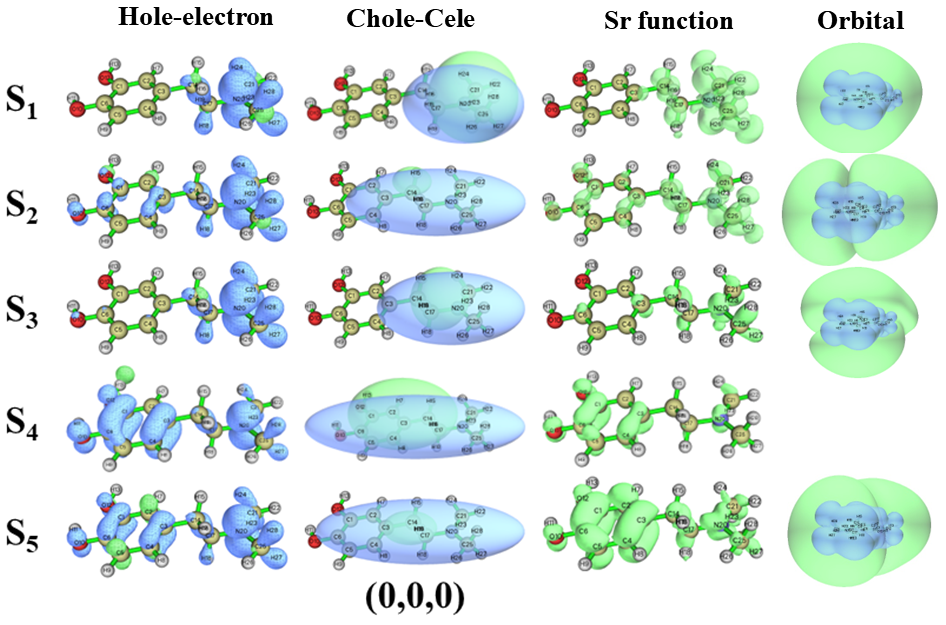


Fig. S7. The hole-electron diagram, Chole-Cele diagram, Sr function diagram and molecular orbital of the five excited states of N,N-dimethyldopamine (0,0,0) conformation. The calculation method and basis set are: CAM-B3LYP/aug-cc-pVTZ.


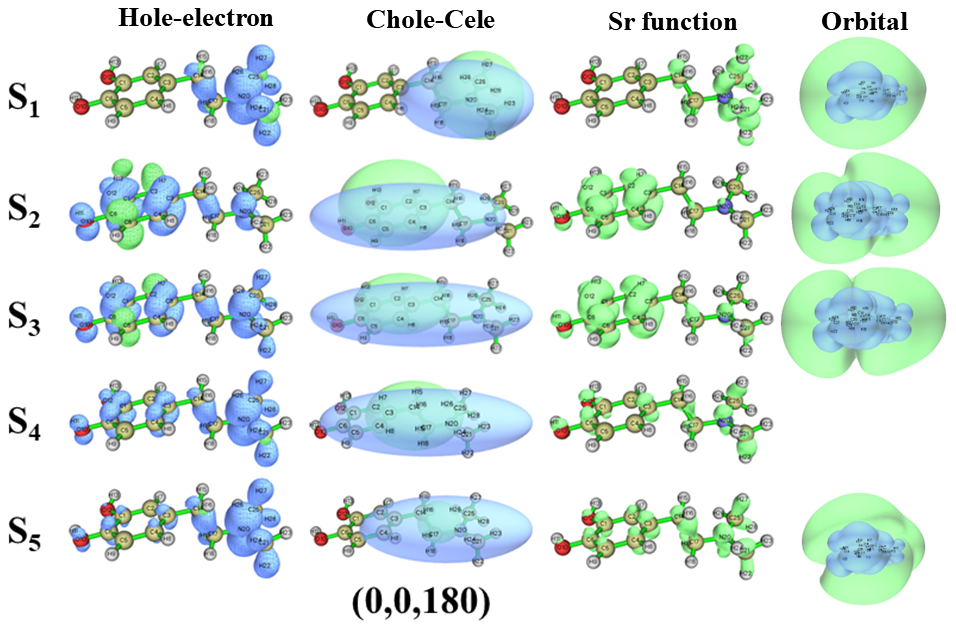


Fig. S8. The hole-electron diagram, Chole-Cele diagram, Sr function diagram and molecular orbital of the five excited states of N,N-dimethyldopamine (0,0,180) conformation. The calculation method and basis set are: CAM-B3LYP/aug-cc-pVTZ.


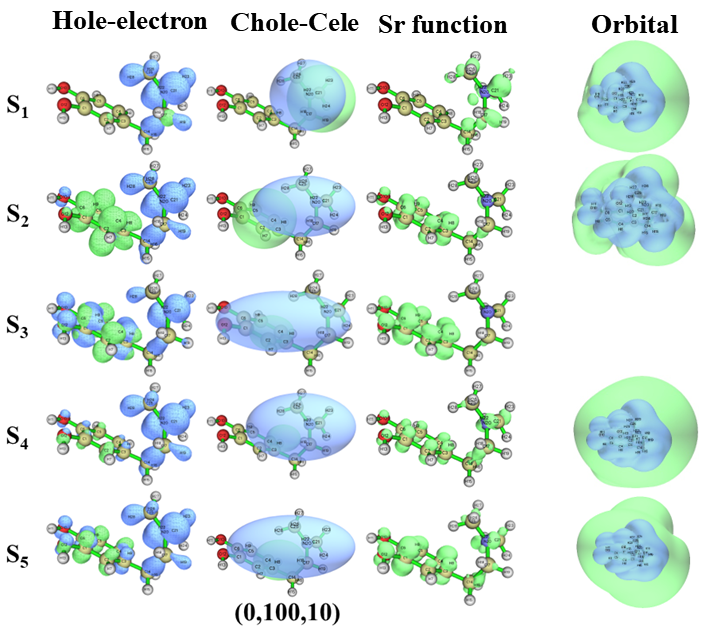


Fig. S9. The hole-electron diagram, Chole-Cele diagram, Sr function diagram and molecular orbital of the five excited states of N,N-dimethyldopamine (0,100,10) conformation. The calculation method and basis set are: CAM-B3LYP/aug-cc-pVTZ.


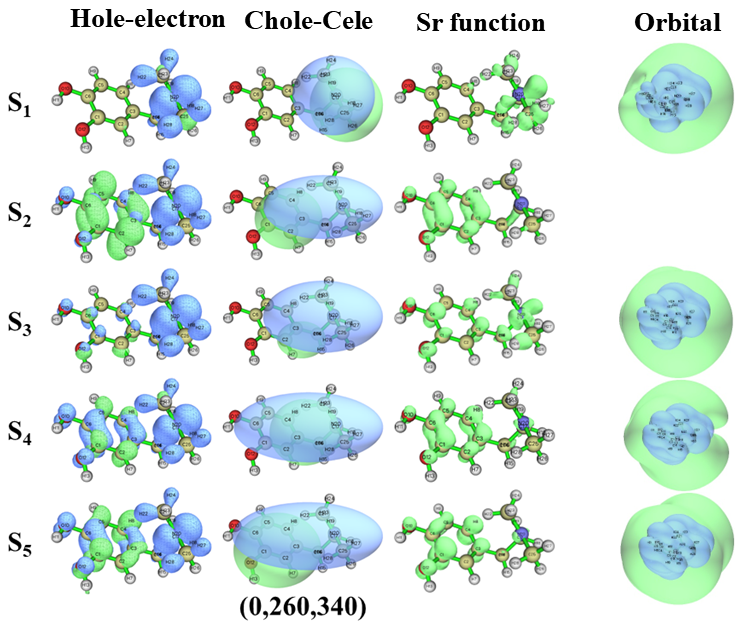


Fig. S10. The hole-electron diagram, Chole-Cele diagram, Sr function diagram and molecular orbital of the five excited states of N,N-dimethyldopamine (0,260,340) conformation. The calculation method and basis set are: CAM-B3LYP/aug-cc-pVTZ.

# S8. Excited state information of N, N-dihydroxydopamine


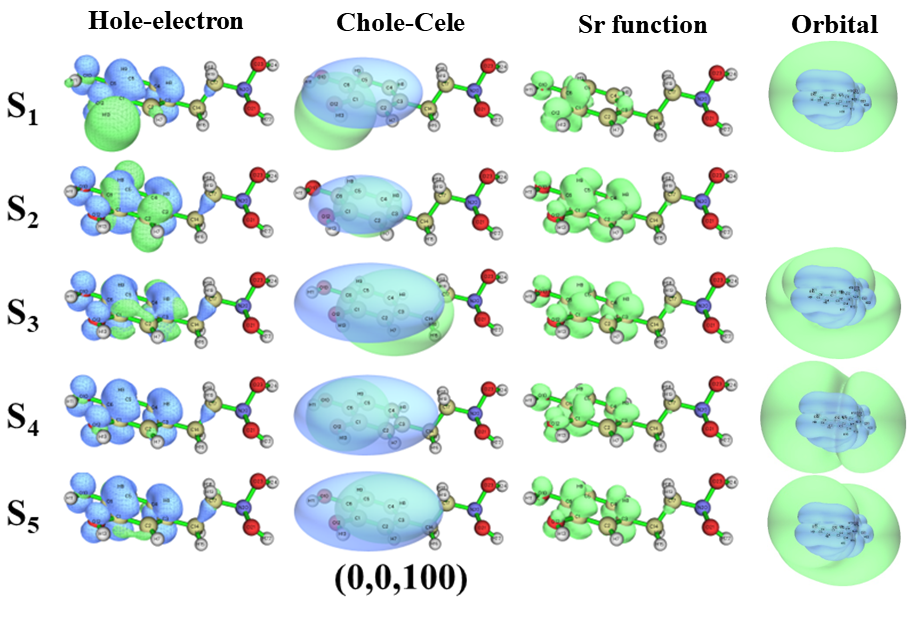


Fig. S11. The hole-electron diagram, Chole-Cele diagram, Sr function diagram and molecular orbital of the five excited states of N,N-dihydroxydopamine (0,0,100) conformation. The calculation method and basis set are: CAM-B3LYP/aug-cc-pVTZ.


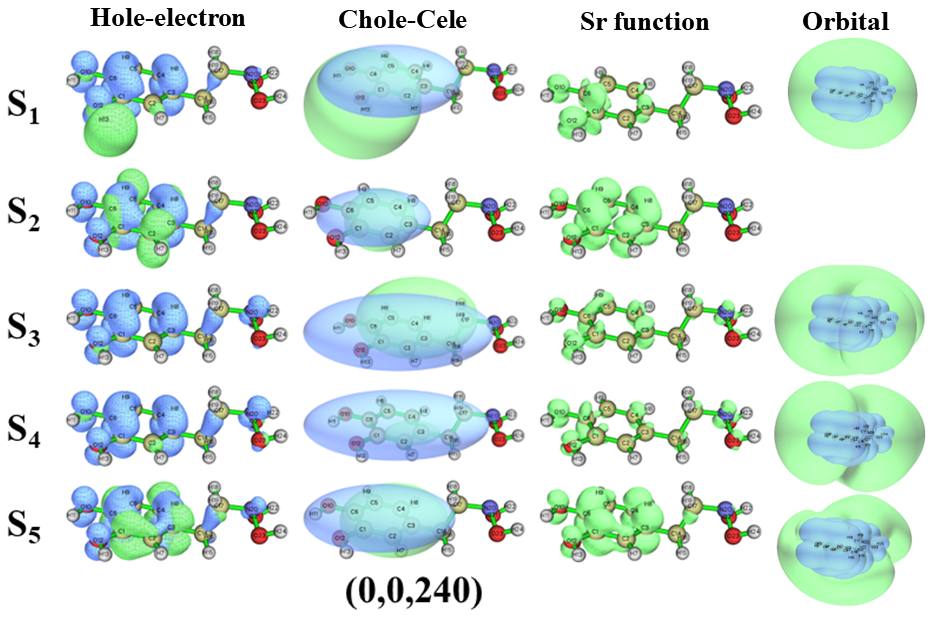


Fig. S12. The hole-electron diagram, Chole-Cele diagram, Sr function diagram and molecular orbital of the five excited states of N,N-dihydroxydopamine (0,0,240) conformation. The calculation method and basis set are: CAM-B3LYP/aug-cc-pVTZ.


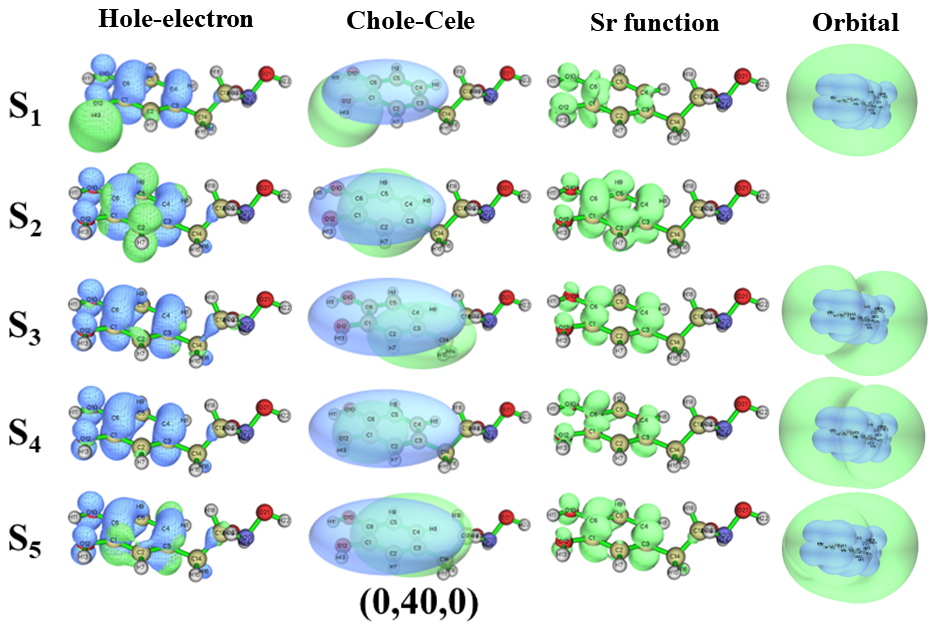


Fig. S13. The hole-electron diagram, Chole-Cele diagram, Sr function diagram and molecular orbital of the five excited states of N,N-dihydroxydopamine (0,40,0) conformation. The calculation method and basis set are: CAM-B3LYP/aug-cc-pVTZ.


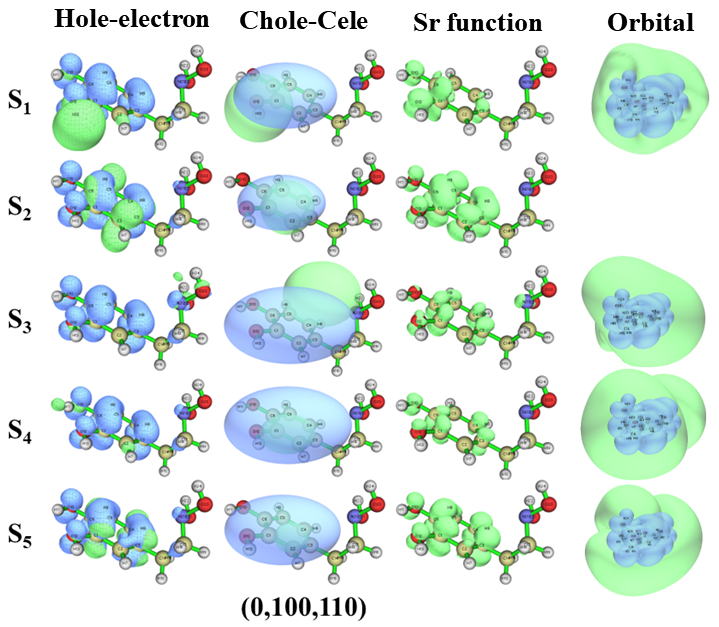


Fig. S14. The hole-electron diagram, Chole-Cele diagram, Sr function diagram and molecular orbital of the five excited states of N,N-dihydroxydopamine (0,100,110) conformation. The calculation method and basis set are: CAM-B3LYP/aug-cc-pVTZ.


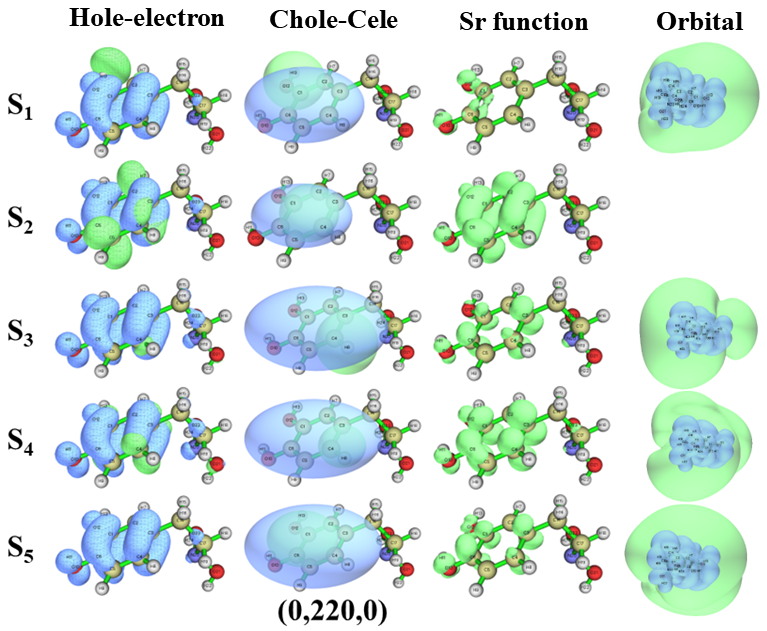


Fig. S15. The hole-electron diagram, Chole-Cele diagram, Sr function diagram and molecular orbital of the five excited states of N,N-dihydroxydopamine (0,220,0) conformation. The calculation method and basis set are: CAM-B3LYP/aug-cc-pVTZ.


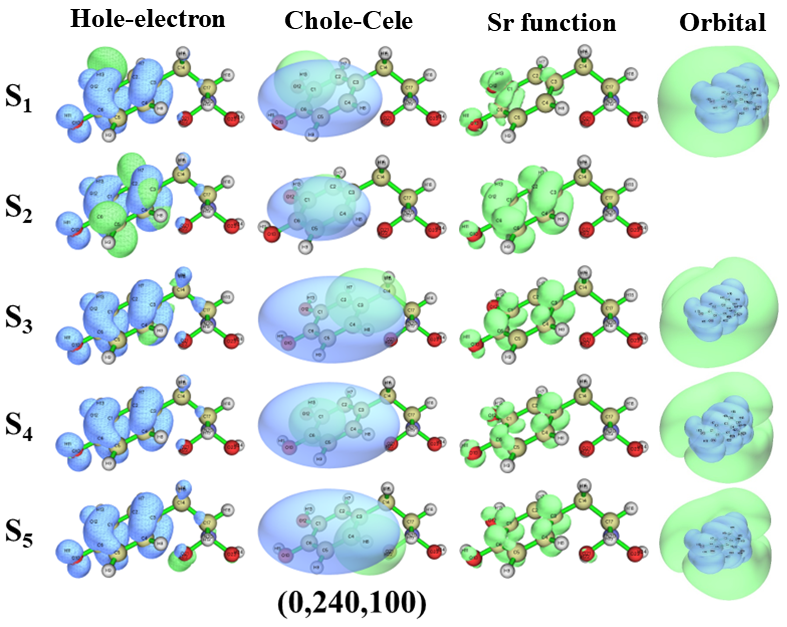


Fig. S16. The hole-electron diagram, Chole-Cele diagram, Sr function diagram and molecular orbital of the five excited states of N,N-dihydroxydopamine (0,240,100) conformation. The calculation method and basis set are: CAM-B3LYP/aug-cc-pVTZ.


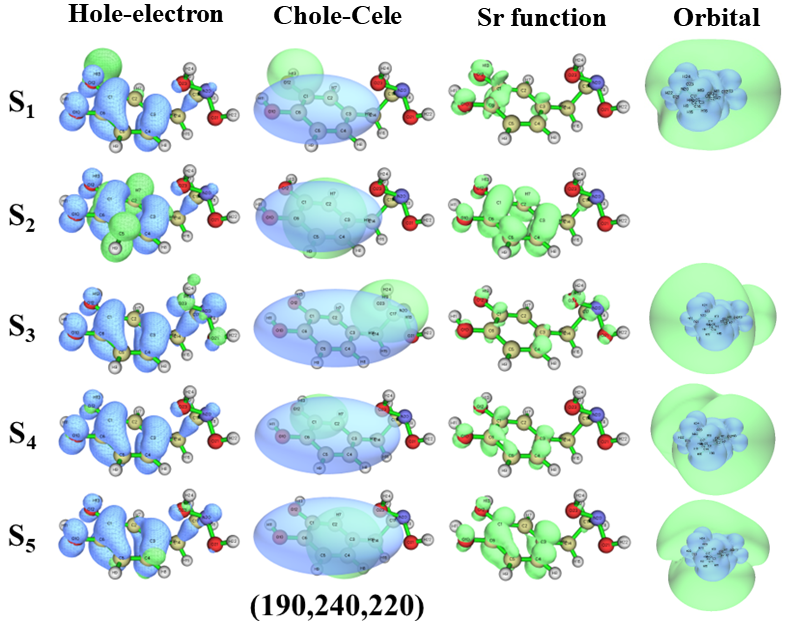


Fig. S17. The hole-electron diagram, Chole-Cele diagram, Sr function diagram and molecular orbital of the five excited states of N,N-dihydroxydopamine (190,240,220) conformation. The calculation method and basis set are: CAM-B3LYP/aug-cc-pVTZ.

# S9. References

[1] T. Yadav, V. Mukherjee, Interpretation of IR and Raman spectra of dopamine neurotransmitter and effect of hydrogen bond in HCl, J. Mol. Struct. 1160 (2018) 256-270, https://doi.org/ 10.1016/j.molstruc.2018.01.066.

[2] J. Giesecke, Refinement of the Structure of Dopamine Hydrochloride, Acta Cryst. B36 (1980) 178-181, https://doi.org/10.1107/S0567740880002798.
